# Colicins of Escherichia coli Lead to Resistance against the Diarrhea-Causing Pathogen Enterotoxigenic E. coli in Pigs

**DOI:** 10.1128/spectrum.01396-22

**Published:** 2022-10-03

**Authors:** Leli Wang, Yuwei Wu, Juan Xu, Qiuyun Huang, Ying Zhao, Sheng Dong, Xuxiang Wang, Xiaoni Cao, Chuni Wang, Anqi Wu, Diao Zhou, Cang Chen, Huansheng Yang, Jianzhong Li, Papadimitriou Konstantinos, Qiang Tu, Gaihua Zhang, Jia Yin

**Affiliations:** a National and Local Joint Engineering Laboratory of Animal Peptide Drug Development, Hunan Provincial Key Laboratory of Animal Intestinal Function and Regulation, Hunan International Joint Laboratory of Animal Intestinal Ecology and Health, College of Life Sciences, Hunan Normal University, Changsha, China; b Laboratory of Food Quality Control and Hygiene, Department of Food Science and Human Nutrition, Agricultural University of Athens, Athens, Greece; c CAS Key Laboratory of Quantitative Engineering Biology, Shenzhen Institute of Synthetic Biology, Shenzhen Institutes of Advanced Technology, Chinese Academy of Sciences, Shenzhen, China; Nanchang University

**Keywords:** *Escherichia coli*, genome sequencing, comparative genomics analysis, secondary metabolite, microbe-pathogen interactions

## Abstract

Gut microbes can affect host adaptation to various environment conditions. Escherichia coli is a common gut species, including pathogenic strains and nonpathogenic strains. This study was conducted to investigate the effects of different E. coli strains in the gut on the health of pigs. In this study, the complete genomes of two E. coli strains isolated from pigs were sequenced. The whole genomes of Y18J and the enterotoxigenic E. coli strain W25K were compared to determine their roles in pig adaptation to disease. Y18J was isolated from feces of healthy piglets and showed strong antimicrobial activity against W25K *in vitro*. Gene knockout experiments and complementation analysis followed by modeling the microbe-microbe interactions demonstrated that the antagonistic mechanism of Y18J against W25K relied on the bacteriocins colicin B and colicin M. Compared to W25K, Y18J is devoid of exotoxin-coding genes and has more secondary-metabolite-biosynthetic gene clusters. W25K carries more genes involved in genome replication, in accordance with a shorter cell cycle observed during a growth experiment. The analysis of gut metagenomes in different pig breeds showed that colicins B and M were enriched in Laiwu pigs, a Chinese local breed, but were scarce in boars and Duroc pigs.

**IMPORTANCE** This study revealed the heterogeneity of E. coli strains from pigs, including two strains studied by both *in silico* and wet experiments in detail and 14 strains studied by bioinformatics analysis. E. coli Y18J may improve the adaptability of pigs toward disease resistance through the production of colicins B and M. Our findings could shed light on the pathogenic and harmless roles of E. coli in modern animal husbandry, leading to a better understanding of intestinal-microbe–pathogen interactions in the course of evolution.

## INTRODUCTION

With the increasing scale of animal husbandry, the disease resistance of pig breeds in modern agriculture is essential for optimal swine production (1). Interestingly, indigenous livestock breeds from some regions have been reported to possess higher disease resistance than commercial breeds (2, 3). For example, the Laiwu pig, an indigenous breed distributed in eastern China, was found to have high disease resistance and excellent meat quality, with characteristics such as good tenderness and bright color (4, 5). The high disease resistance might be related to the coevolution of native breeds of livestock with their environment, and such breeds have the highest likelihood of survival during disease outbreaks (6).

It is well known that wild animals are able to subsist on pathogen-infected food and show immunity to various diseases (7, 8). A recent study revealed that a protease, termed WBT001, from the gut microbiota of wild carrion eaters could metabolize bacterial toxins (8). Hu et al. (2) revealed that the Congjiang miniature piglet (a Chinese native pig breed) has stronger resistance to early-weaning stress-induced diarrhea than commercial crossbred piglets. This property of Congjiang miniature piglets was related to the gut microbiota-derived bacteriocin, gassericin A (2). These findings indicated that native-breed animals may have retained some beneficial gut microbes and metabolites involved in disease resistance during their evolution (9, 10).

Gut microbiotas and their bioactive metabolites in pigs have been studied for decades. The exact mechanisms involved in interactions between the microbiome and the gut physiology of pigs are diverse and have not been resolved in detail yet (11, 12). Among the members of the gut microbiota, Escherichia coli is one of the first bacteria to colonize the gastrointestinal tract of the host at birth, which paves the way for the establishment of *Bifidobacterium* and *Bacteroides* (13). E. coli is composed of diverse strains, some of which are notorious pathogens. Uropathogenic E. coli can cause host urinary tract infections (14), avian-pathogenic E. coli (APEC) can cause colibacillosis in poultry (15), neonatal meningitis E. coli (NMEC) can cause meningitis in newborn infants (16), and enterotoxigenic E. coli (ETEC), adherent invasive E. coli (AIEC), enteropathogenic E. coli (EPEC), enteroaggregative E. coli (EAEC), enterohemorrhagic E. coli (EHEC), and Shiga toxin-producing enteroaggregative E. coli (StxEAEC) can all cause diarrhea in mammals (17). Of these pathogens, ETEC is mainly responsible for neonatal diarrhea, postweaning diarrhea, and edema disease in the pig industry. In previous studies, ETEC W25K rapidly colonized the intestine after infection and secreted exotoxins, including heat-labile enterotoxin (LT) and heat-stable enterotoxin (STa/STb), leading to intestinal dysregulation and piglet diarrhea (18–20).

One question that arises is whether there are microorganisms in the animal gut that are selected to antagonize pathogenic microorganisms during long-term evolution. Interestingly, the majority of E. coli strains that colonize the healthy human and mammalian gut are nonpathogenic commensals or even probiotics. For example, E. coli Nissle 1917 has already been applied to the treatment of multiple gastrointestinal diseases, including diarrhea, uncomplicated diverticular disease, and inflammatory bowel disease (21). These indigenous intestinal bacteria also play an important role in protection against pathogens. Several important underlying mechanisms are responsible for the antagonistic effects of the indigenous bacteria against pathogens, including immunomodulation, competitive exclusion for adhesion sites and nutritional sources, enhancement of intestinal barrier function, and secretion of antimicrobial substances, but their specific modes of action are not very well understood (22). Furthermore, the presence of both nonpathogenic and pathogenic bacteria within the same genus indicates that their genomic characteristics may vary, which leads to differences in their phenotypic characteristics and functions.

This study was conducted to investigate the heterogeneity of E. coli strains from pigs and to determine whether the production of secondary metabolites could improve adaptability of hosts toward disease resistance in the course of evolution. In the study, an E. coli strain Y18J was isolated from feces of healthy piglets, and the strain showed strong antimicrobial activity against ETEC W25K. We sequenced and analyzed the complete genomes of both Y18J and ETEC W25K to investigate the potential genomic features of the observed interaction phenomenon. Genome mining revealed that the antagonistic activity of Y18J against W25K was related to genes for colicins B and M. The distributions of functional genes in Y18J and W25K among boars and Duroc and Laiwu pigs were different. In Laiwu pigs, the distribution frequencies of colicins B and M were significantly higher than the frequencies of pathogenic factors, including LT and STa/STb (*P = *3.76E−05). Colicin B and colicin M may have been retained against pathogenic bacteria during the evolution of native pig breeds.

## RESULTS

### Bacteria targeted screening, antibiotic resistance, and sequencing.

**FIG 1:**
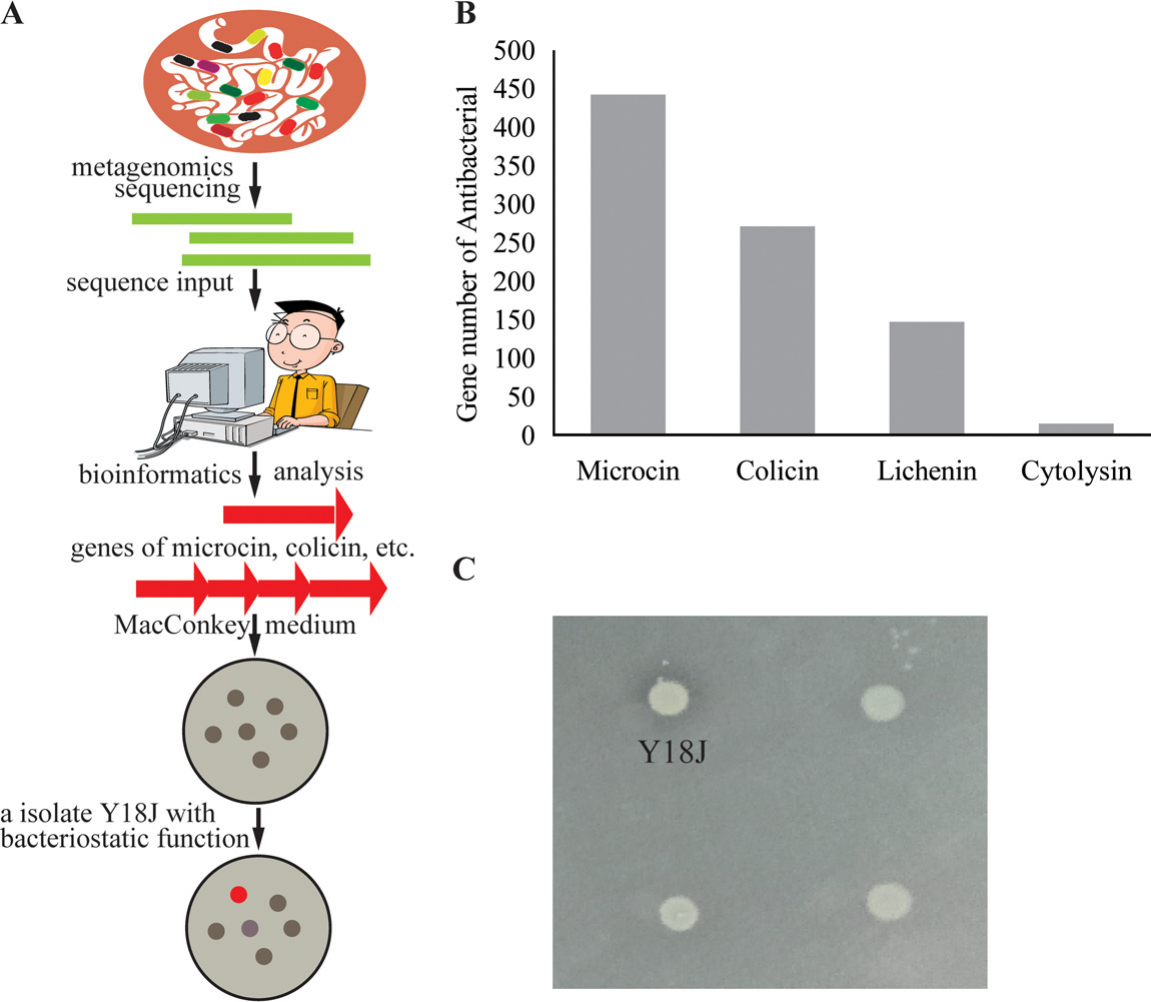
Bioinformatics-based screening of targeted bacteria with antibacterial activity. (A) Flowchart of targeted screening. (B) Numbers of genes with antibacterial activity in the pig metagenome. (C) Inhibitory effects of wild bacterial strain Y18J against the pathogen W25K.

The nontargeted screening of bacteria during the functional characterization of the gut microbes is time-consuming and laborious. Therefore, we have built a bioinformatics-based screening strategy for targeting specific bacteria (Fig. 1A). Briefly, the metagenomics data in a previous report (9) were downloaded, and bioinformatics analysis revealed that the antibacterial-related genes encoded microcin, colicin, lichenin, and cytolysin (Fig. 1B). Given that colicins and some microcins are encoded by E. coli, a custom medium was used for its isolation. Using MacConkey agar medium supplemented with antibiotics and antifungals, hundreds of E. coli isolates were obtained based on colony morphology and 16S rRNA gene sequence from the feces of healthy piglets. Nystatin and erythromycin were added to inhibit the growth of fungi and Gram-positive bacteria, respectively. In the antimicrobial assay, one bacterium, Y18J, was selected for further analysis, as it showed the highest antimicrobial activity against the ETEC strain W25K (Fig. 1C), a diarrheagenic strain in piglets (23).

Antibiotic resistance genes (ARG) are mainly acquired through horizontal gene transfer (HGT) (24) and natural mutation acquisition (25). Antibiotic resistance of indigenous gut bacteria can provide a competitive advantage during evolution. Thus, the susceptibilities of Y18J and W25K to 26 antibiotics from seven different classes were also evaluated (see Tables S1 and S2 in the supplemental material). Compared to W25K, Y18J was sensitive to more antibiotics, including kanamycin, gentamicin, and hygromycin. Such antibiotic sensibility could be used for subsequent gene knockout experiments.

The genomes of Y18J and W25K were sequenced, and their features are summarized in Table S3 and Fig. S1. Moreover, the presence of drug resistance genes in the genome of Y18J was analyzed using the Comprehensive Antibiotic Resistance Database (CARD) (26), and 54 drug resistance genes were identified. A beta-lactamase (gene 3985; *CMY-47*), an ABC efflux pump (gene 1919; *msrB*), a chloramphenicol exporter (gene 2926; *mdfA*), and a multidrug and toxic compound extrusion exporter (gene 1712; *mdtK*) were predicted to be related to resistance to penicillins (27), erythromycin (28), chloramphenicol (29), and norfloxacin (30), respectively (Table S4 and Data Set S1). Other ARGs, such as an efflux pump complex (*acrD*, *rosA*, *rosB*, *mdtN*, *mdtO*, *mdtP*, and *acrB*) and fluoroquinolone (*patA*) could be involved in acquiring tolerance to other antibiotics (Table S1). These drug resistance genes in the Y18J genome would facilitate its survival in the presence of antibiotics.

### Phylogeny and genes involved in virulence.

**FIG 2:**
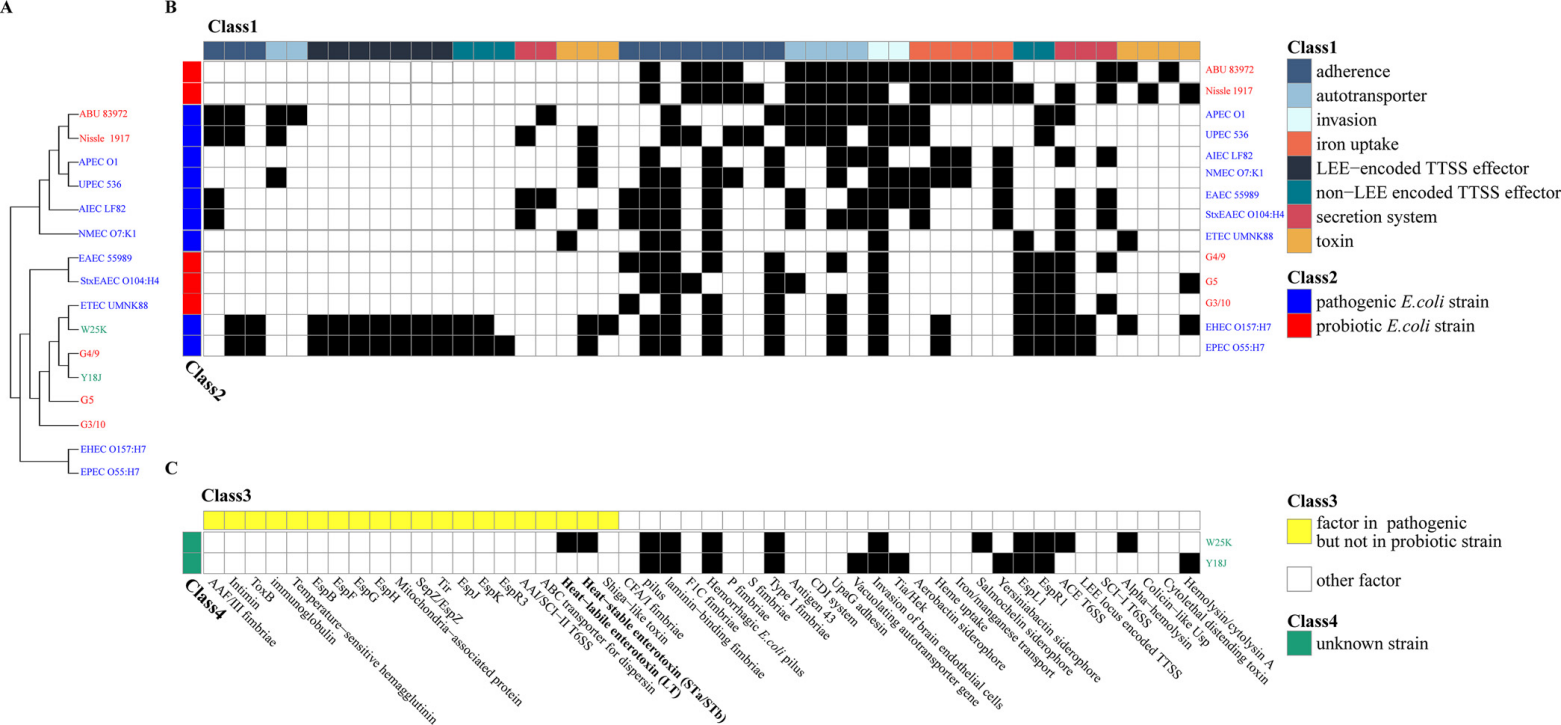
Phylogeny and virulence factors present in Y18J and W25K. (A) Neighbor-joining phylogenetic tree obtained from g*yrB* gene sequences. The bootstrap consensus tree inferred from 500 replicates was taken to represent the evolutionary history of the taxa analyzed. Branches corresponding to partitions reproduced in less than 50% of bootstrap replicates were collapsed. (B) Virulence-associated genes were identified using VFDB in known pathogenic E. coli strains and probiotic E. coli strains. The bacteria in red are commercial probiotics. The bacteria in blue are pathogens. (C) Virulence-associated genes were identified using VFDB in W25K and Y18J. Black squares indicate the presence of factors; white squares indicate the absence of factors.

A neighbor-joining phylogenetic tree was created with the *gyrB* gene sequences (Fig. 2A). A total of 16 E. coli strains, including both probiotic and pathogenic bacteria, were analyzed. Based on the phylogenetic analysis, the results revealed that the Y18J was closely related to W25K and the probiotic strain G4/9.

To understand the virulence potential of Y18J, we compared the virulence factors using the Virulence Factor Database (VFDB) for Y18J, W25K, and 14 other E. coli strains (Fig. 2B and C) (31). It was revealed that Y18J contained only a toxin, namely, hemolysin/cytolysin A (Fig. 2C). W25K contained LT and STa/STb, which could induce diarrhea in pigs (32, 33). Noteworthily, we found that the enterotoxins LT and STa/STb also existed in the other pathogenic E. coli strains but not in probiotic strains (Fig. 2B and C).

Interestingly, some genes involved in adhesion were identified in all analyzed probiotic strains, including Y18J. In order to further confirm the biosafety potential of Y18J *in vivo*, the diarrhea rate in mice was evaluated after gavage feeding with Y18J and W25K. Diarrhea occurred after gavage feeding of pathogenic W25K at an average rate of 44.67% but did not occur after gavage feeding of strain Y18J (*P < *0.05) (Fig. S2), which suggested that Y18J is a nonpathogenic E. coli strain.

### Comparative genomic analysis.

**FIG 3:**
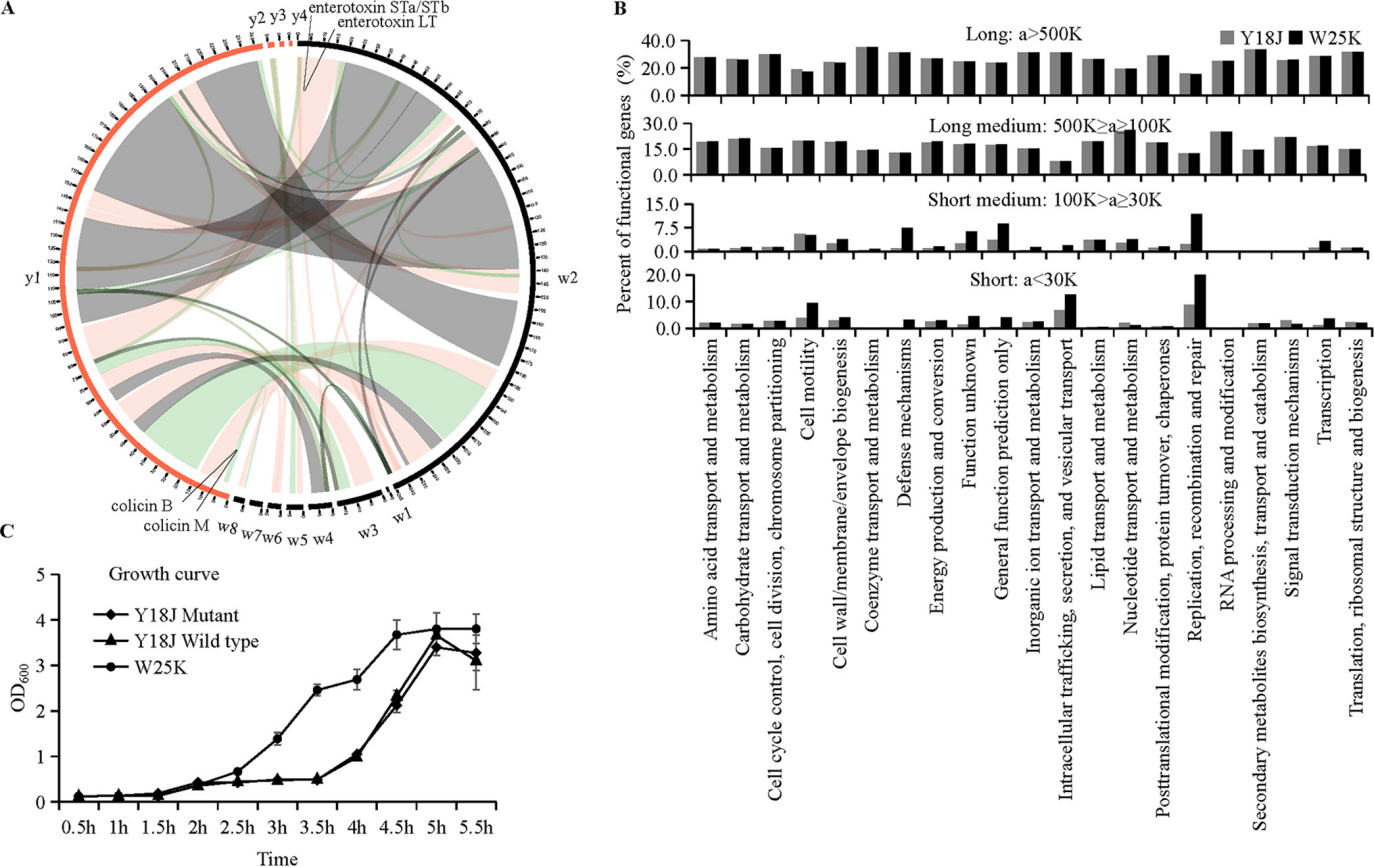
Homology genomic and growth curve comparisons between Y18J and W25K. (A) Circos plot showing homology regions between Y18J (including y1, y2, y3, and y4) and W25K (including w1, w2, w3, w4, w5, w6, w7, and w8). The homology regions are shaded in different colors. (B) COG function annotation of the different homology regions according to the length of homology region. The length of homology regions is represented by the letter “a.” (C) Growth curves for Y18J (wild type and mutant) and W25K. The Y18J mutant is a strain in which colicins B and M were knocked out. The optical density at 600 nm (OD_600_) was measured from a starting OD_600_ of 0.085. Data are means and standard deviations (SD) from three independent experiments.

Both strains Y18J and W25K were isolated from pigs, and their phylogenetic relationship was close. To assess the overall genetic differences between Y18J and W25K, we proceeded with a comparative genomics analysis at the whole-genome level. The Circos plot revealed that Y18J and W25K have genomic regions of high homology (Fig. 3A and Table S3). In order to better evaluate the gene ontology and function classification of these strain-specific genes, we performed Gene Ontology (GO), Cluster of Orthologous Groups of proteins (COG), Carbohydrate-Active enZYmes Database (CAZymes) and Kyoto Encyclopedia of Genes and Genomes (KEGG) enrichment analyses. Based on the four databases, we found that the number of genes in W25K was larger than that in Y18J for the majority of gene functions (Fig. S3 to S5 and Data Sets S2 to S5), especially for the virus-related genes. The Y18J strain possesses only 46 and 43 genes for the virion and virion part genes, respectively, while W25K possesses 105 and 97 genes (Fig. S4 and Data Set S4). However, there were some exceptions. The numbers of genes for molecular transducer activity, receptor activity, and translation regulation regulator activity from GO terms (Fig. S4 and Data Set S4) and glycosyl transferases and carbohydrate esterase from CAZymes classes (Fig. S5 and Data Set S5) in Y18J were higher than in W25K. Additionally, the number of genes for secondary-metabolite biosynthesis, transport, and catabolism from COG function classification (Fig. S3A and Data Set S2) and the metabolism of terpenoids and polyketides from KEGG classification (Fig. S3B and Data Set S3) in Y18J was slightly higher than in W25K.

Due to the fact that the overall levels of various functional genes are almost identical (Fig. S3, Fig. S4, Fig. S5 and Data Set S2 to 5), we focused on homology regions of Y18J and W25K, which may contain a large number of conserved genes in the evolution of bacteria (34, 35). According to the average length of homology regions (Fig. 3A), these homology regions were divided into four types, including the long region, medium long region, medium short region, and short region (Fig. 3B). We found that in the genetic regions of <100 kb, the proportion of genes which were related to replication, recombination, and repair was higher in W25K than in Y18J, which may explain why W25K grows faster than Y18J (Fig. 3C). Inversely, among the majority of homologous regions of >100 kb, there was no difference between the proportions of functional genes of Y18J and W25K in the main metabolic pathways. These findings were in agreement with the close relatedness of Y18J to the pathogen ETEC W25K according to the phylogenetic analysis (Fig. 2A). The genes involved in defense mechanisms, cell motility, unknown functions, transcription, and general functions were present in a higher proportion in W25K than in Y18J (Fig. 3B).

### Secondary-metabolism gene clusters and microbe-microbe interactions.

**FIG 4:**
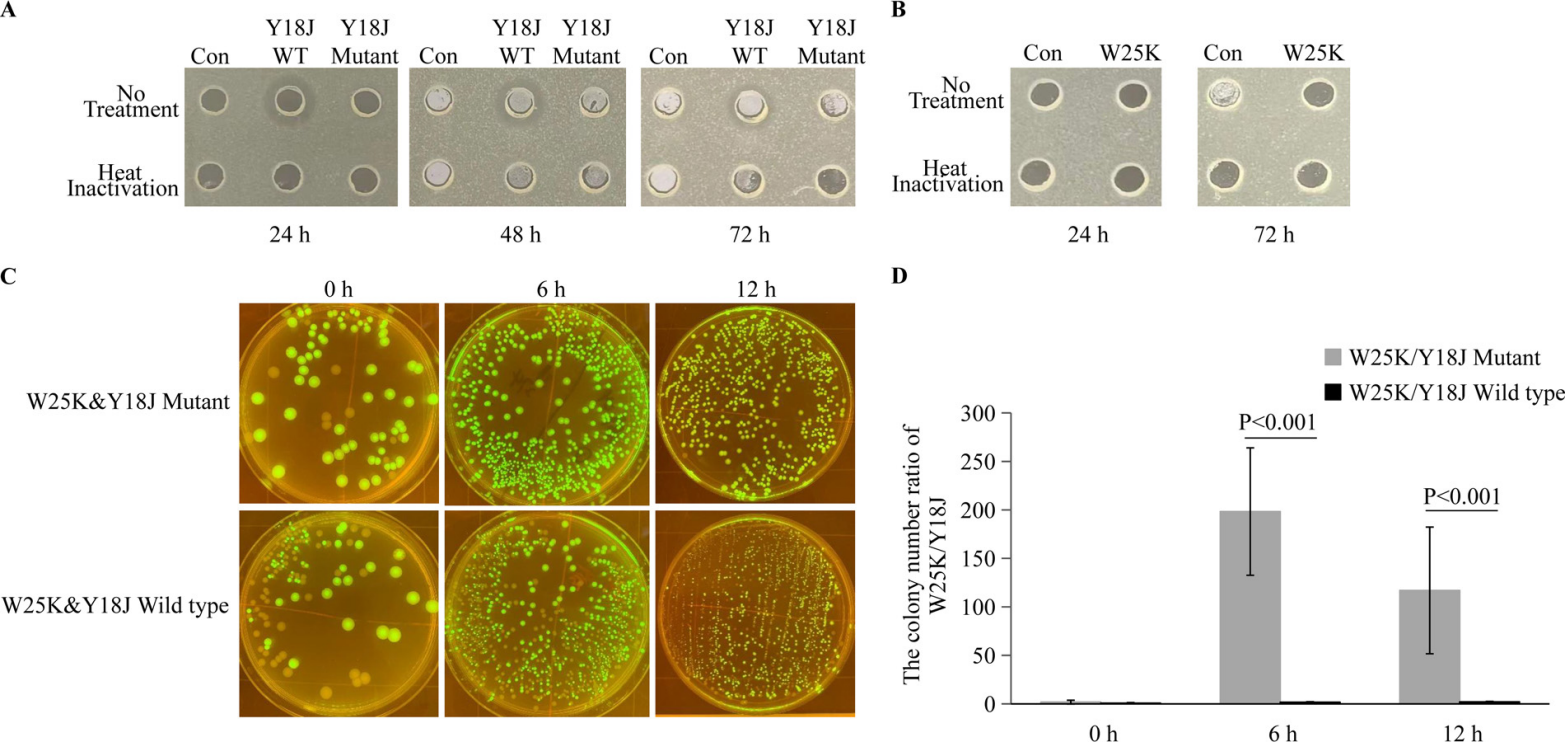
Modeling of the microbe-microbe (W25K-Y18J) interactions *in vitro*. (A) Inhibitory effects of the Y18J wild-type and mutant strains at different growth times against the pathogen W25K. The antimicrobial products in the growth medium were inactivated with and without heat treatment. Con, control group treated with LB medium. (B) Inhibitory effects of W25K against Y18J at different growth times. (C) Y18J (wild-type or mutant) and W25K colonies at different coculture times. The colony without fluorescence is Y18J, and the fluorescent colony is W25K with a GFP expression plasmid. (D) Ratios of numbers of colonies of W25K and Y18J.

Members of the gut microbiota synthesize a wide variety of bioactive secondary metabolites in order to compete for nutrition and living space. These substances might not be necessary for the normal growth of these bacteria, but they constitute an essential molecular resource pool for colonizing the host intestine (36). Therefore, the genome sequences were analyzed by antiSMASH, and the result showed that there were four and two biosynthetic gene clusters (BGCs) in the Y18J and W25K genomes (Table S5), respectively. BGC1 of Y18J was predicted to have 18% sequence similarity to microcin L genes. Bioinformatics analysis indicated that BGC1 contained the operons for the bacteriocins colicin B and colicin M (Fig. S6A), and these operons were almost always found in close proximity on the same large conjugative plasmid. Colicin B kills sensitive cells by forming ion channels that depolarize the cytoplasmic membrane, while colicin M is unique in its killing action, leading to lysis of target cells by inhibiting biosynthesis of peptidoglycan and lipopolysaccharide O antigen (37). In order to study the antimicrobial mechanism of Y18J against W25K, we first knocked out the colicin B and M operons of Y18J using Red/ET recombineering (Fig. S6B). The deletion of the colicin B and M operons did not impact the growth of Y18J (Fig. 3C). The antagonistic activities of wild-type or mutant Y18J, with or without heat inactivation, were first tested against W25K using the agar well diffusion assay (38). The results showed that the antagonistic activity disappeared in the mutant or when the growth medium was heat inactivated (Fig. 4A). There was no antagonistic activity of W25K against Y18J (Fig. 4B). In addition, the antagonistic activity appeared again against W25K in the colicin B and M complementation strains, and the antagonistic activity of wild-type Y18J was stronger than that of the colicin B and M complementation strains (Fig. S7), which suggests that colicins B and M are responsible for the antagonistic activity against W25K.

To gain a better understanding of the interaction between Y18J and W25K, a simplified coculture model was constructed to investigate the potential microbe-microbe interactions. A green fluorescent protein (GFP) expression plasmid was introduced into the pathogenic strain W25K, which was subsequently cocultured with wild-type or mutant Y18J. The broths from different coculture times were plated on agar plates after dilution, and the colonies of Y18J and W25K were counted (Fig. 4C). As determined by comparing the colony number ratios of W25K and Y18J mutant strains, the growth of W25K was inhibited by wild-type Y18J (Fig. 4D). These results suggested that the production of colicins B and M in Y18J could play an important role in preventing the invasion of pathogenic W25K in piglets.

### Prevalence of pathogenic factors and antagonistic factors.

In the process of long-term evolution, hosts have developed different survival strategies to resist E. coli (39–41). For this reason, we investigated the relationships between colicin B and colicin M in different pig breeds. Wild boars, Duroc pigs, and Laiwu pigs, which respectively represent pigs in free living conditions, in standard commercial farms, and in an indigenous environment in eastern China (42, 43), were selected to compare the distribution of known pathogenic factors and new antagonistic factors. The prevalence of pathogenic factors and antagonistic factors in microbial genome resources of boars and Duroc and Laiwu pigs was calculated. For W25K, we used STa/STb (W_1 gene) and LT (W_2 gene) as markers. For Y18J, colicin B (Y_1 gene) and colicin M (Y_2 gene) were used as markers, prior to the investigations of these four genes’ prevalence in different pigs.

**FIG 5:**
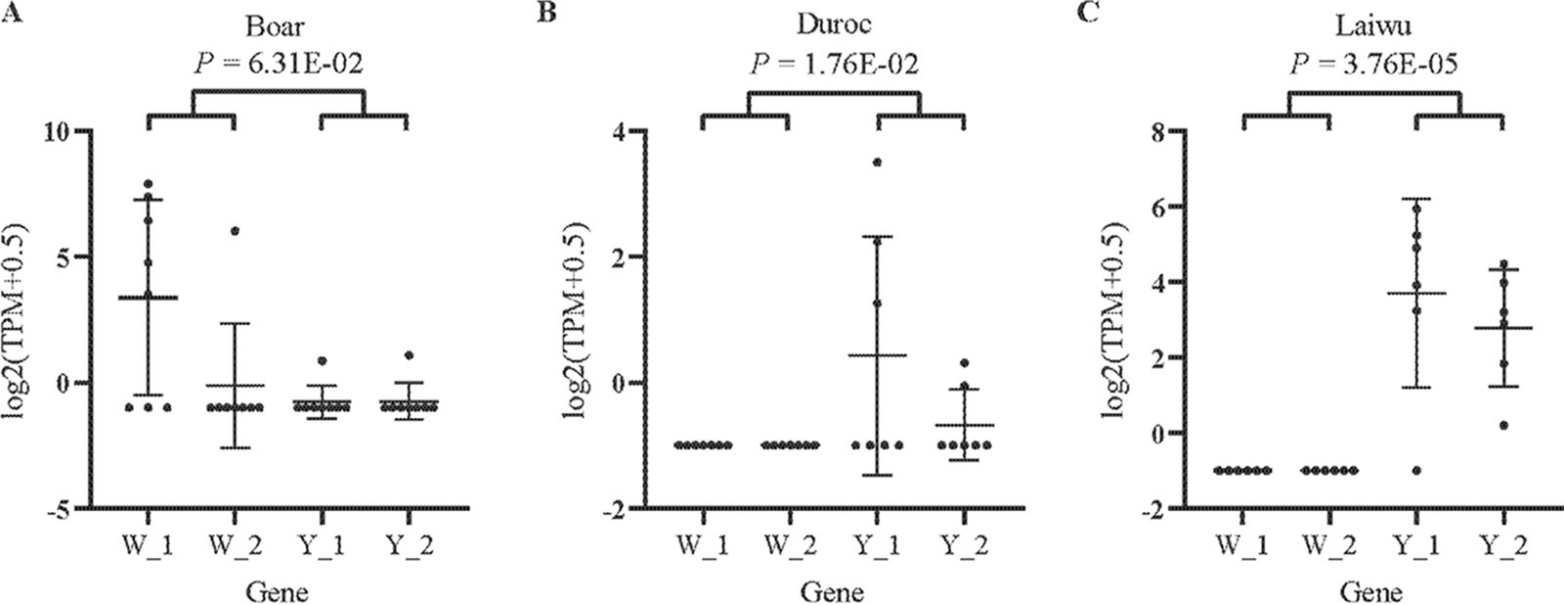
Levels of pathogenic and antagonistic factors from different origins. (A) Boars; (B) Duroc pigs; (C) Laiwu pigs. The pathogenic factors encoded by W_1 and W_2 in W25K are a heat-stable enterotoxin and a heat-labile enterotoxin, respectively. The antagonistic factors encoded by Y_1 and Y_2 in Y18J are colicin B and colicin M, respectively. Statistical analyses were performed using the Mann-Whitney U test (R version 3.5; a *P* value of <0.01 was considered statistically significant).

The Salmon method was used to calculate the transcripts per million (TPM) for all boars, Duroc pigs, and Laiwu pigs (Fig. S8). Based on the TPM values, 8 of 14 samples from boars (Fig. S9) and 7 of 10 samples from Duroc pigs (Fig. S10) were selected by Spearman’s nonparametric correlation analysis and clustering. In boars, the frequencies of W_1 and W_2 were slightly higher than those of Y_1 and Y_2 (*P = *6.31E−02). In Duroc pigs, a commercial breed raised in extensive pig farms, there was no difference between the frequencies of two pathogenic factors and the frequencies of two antagonist factors (*P = *1.76E−02). Surprisingly, for Laiwu pigs, which is an indigenous breed distributed in northeast China that exhibits excellent meat quality (4), the frequencies of antagonist factor genes Y_1 and Y_2 were significantly higher than the frequencies of pathogenic factor genes W_1 and W_2 (*P = *3.76E−05) (Fig. 5 and Data Sets S6 and S7).

## DISCUSSION

Pig domestication first occurred in Europe and Asia from wild boar populations nearly 10,000 years ago (44). With the evolution and selective breeding of domestic animals, pigs have functioned as a major source of protein for humans (45). Different types of native pigs have experienced substantial genetic differentiation after a long period of evolution (46). Gut microbes play an important role in host health, and they also evolve along with the changes in the living environment of hosts. E. coli from the mammalian gut can be commensal and/or the cause of various infectious diseases (47). Y18J, used in this study, is a nonpathogenic indigenous bacterium isolated from a healthy pig, while W25K can cause diarrhea in piglets. Genome sequencing revealed that the two strains shared a high degree of homology (Fig. 3A). We searched for the factors which may cause the two bacteria to adapt to the same host and perform different biological functions. The biological functions are consistent in the highly conserved regions, which account for most of the gene functions (Fig. 3B, top two panels), but the small proportion of genes in the poorly conserved regions are related to different biological functions (Fig. 3B, bottom two panels), which might cause the two bacteria to evolve different host adaptation mechanisms.

### Nontoxicity and stress resistance in the indigenous bacterium.

For an indigenous bacterium to survive in its host intestine, it should have at least two characteristics. (i) The bacteria should not cause disease in the host. To understand the virulence potential of Y18J, we analyzed the list of virulence factors in W25K, Y18J, and other E. coli strains, including known pathogenic and probiotic strains, using the VFDB (31) (Fig. 2B and C). We found that W25K possessed the enterotoxins LT and STa/STb, which are also encoded in the genomes of known pathogenic E. coli strains (Fig. 2C). LT and STa/STb have been documented as important factors associated with diarrhea diseases in piglets, which can disrupt intestinal fluid homeostasis and cause hypersecretion of fluid and electrolytes through activation of adenylate cyclase (by LT) or guanylate cyclase (by STa) in the small intestinal mucosal cells (48). In addition, some of the genes involved in adhesion were identified in Y18J (Fig. 2C). It is well known that adhesion is essential even for commensal and probiotic bacteria to promote colonization and avoid elimination from the host (49). We further confirmed the lack of pathogenicity of the Y18J strain *in vivo*. In contrast to W25K, the Y18J did not cause diarrhea in the mice model (Fig. S2).

(ii) Indigenous bacteria should have better resistance to stress, such as antibiotics and competition from other intestinal microbes. Antibiotics, such as penicillins, are widely used in the swine industry (50, 51). We tested the sensitivity of Y18J to 26 antibiotics (Table S1) and found that it was resistant to penicillins, erythromycin, chloramphenicol, and norfloxacin. We further analyzed the presence of drug resistance genes using the CARD (26) and identified 54 different drug resistance genes (Table S4). Among these resistance genes, *CMY-47*, *msrB*, *mdfA*, and *mdtK* might be responsible for resistance to penicillins, erythromycin chloramphenicol, and norfloxacin (27–30), respectively.

### Competitive advantages of antibacterial substances.

ETEC is a leading cause of infectious diarrhea in piglets (18, 52). LT and STa/STb are identified as main virulence factors in ETEC (53, 54). In this study, we isolated the nonpathogenic bacterium Y18J originally from healthy pig feces, and it showed strong antimicrobial activity against pathogenic W25K (Fig. 1C). The antiSMASH analysis revealed that there were four and two secondary BGCs in the genomes of Y18J and W25K (Table S5), respectively. Secondary metabolites are products that are not essential for the growth of a bacterium but are produced to confer a selective advantage to it, for example, by functioning as defense compounds, including antibacterial substances or signaling molecules in ecological interactions (55). Indigenous intestinal bacteria in a host play an important role in protection against colonization by pathogens (56), through several mechanisms, including the secretion of antibacterial substances, competitive metabolic interactions, localization to intestinal niches, and induction of host immune responses. One of the antagonistic mechanisms of Y18J is the production of colicin B and colicin M (Fig. 4A, C, and D). Colicin B and colicin M are the most common colicins and are usually encoded adjacently on the same plasmid in E. coli (37). However, the colicin B and colicin M gene operons are located in the chromosome of Y18J. An integrase was found in close proximity to the colicin B and M operons (Fig. S6A), which suggested that this operon may have migrated from a conjugative plasmid by HGT during the evolution of the strain.

At both the neonatal and the postweaning stages, piglet diarrhea usually leads to a high level of mortality in nursery beds and seriously challenges livestock production worldwide (57). One factor in the development of diarrhea is the exposure of piglets to pathogens such as ETEC. In response to infectious diseases in livestock, the common strategy in the past was antibiotic therapy or prevention. However, the increase in multidrug-resistant microorganisms compromised the therapies for a growing number of infectious diseases and threatens public health worldwide (58). Currently, it is prohibited to add antibiotics in livestock and poultry feed in many countries and regions. In the future, it could be a better strategy to improve the immunity or resistance of animals to pathogens. Our study provides evidence that the indigenous gut bacterium could antagonize pathogenic ETEC from pigs.

### Survival strategy of pathogenic bacteria.

Pathogens have evolved strategies to overcome competition by the indigenous intestinal microbes (59). There was no antagonism of the pathogenic strain W25K against the indigenous gut microbe Y18J (Fig. 4B). However, W25K grew faster than Y18J, as demonstrated by the observation that W25K and Y18J entered log-phase growth at 2.5 h and 3.5 h, respectively (Fig. 3C). The fast growth of W25K could endow it with the ability to escape the attack of indigenous gut microbes (60).

In the COG function annotation (Fig. 3B), the percentages of genes with functions including replication, recombination, repair, and defense mechanisms are higher in W25K than in Y18J. Previous studies revealed that replication-associated gene dosage correlates strongly with high growth rate in bacteria (61). In a competitive environment, fast and selfish growth is an effective evolutionary strategy for bacteria to survive (60, 62). Bacterial adaptation also could be acquired by the acquisition of novel traits through HGT. Most mobile genetic elements are in hot spots, and many hot spots exhibit frequent homologous recombination at flanking core genes, which suggests that homologous recombination and HGT are tightly linked in genome evolution (63). Some bacteria have evolved defense mechanisms that directly inhibit the ability of other bacteria to survive, such as the ability to form biofilms, to alter intracellular materials and to regulate genes to protect themselves from bactericides (64). The interplay between the indigenous intestinal microbes and pathogens is essential to control infection and disease. Understanding indigenous intestinal microbe-pathogen interactions may lead to new therapeutic approaches to treating infectious diseases in animal husbandry (60).

In this study, we used microbial resources to investigate the distribution of known pathogenic and new antagonistic factors. We focused on wild boars, Duroc pigs, and Laiwu pigs, which represent pigs in free living conditions, standard farm-raised pigs in the modern commercial pig industry, and indigenous pigs from eastern China, respectively (42, 43). The distribution of genes for pathogenic factors and antagonistic factors is similar in wild boars and Duroc pigs. However, the high frequencies of the antagonistic factors colicins B and M in the gut microbiomes in Laiwu pigs demonstrate that the secondary-metabolite BGCs may involve adaptive features in pigs that help them cope with E. coli being widely distributed in living environments. Persistent ETEC might have put selective pressure on the native microbial population in indigenous hosts, which led to the emergence, propagation, and persistence of antagonistic factors (65). Laiwu pigs were also reported to possess resistance to additional infectious diseases, including porcine circovirus type 2 infection, through the increased expression levels of mannose receptor C type 1 in the liver, kidney, and mesenteric lymph nodes (66). Moreover, compared with that in domesticated animals, the prevalence of the W_1 gene and W_2 gene in wild boars seems high, which might be explained by the increase in a wide range of natural habitats and the frequent exposure to various pathogens (67). Another factor probably involved in the high frequencies of LT and STa/STb in wild boars is that wild animals often act as reservoirs of pathogens or virulence-related genes, with a risk of transmission to humans and livestock (68–70).

### Conclusions.

In this study, we present high-quality genome assemblies and analysis of E. coli Y18J isolated from pig feces. We established a genomic basis for further studying intestinal microbe-microbe interaction. Comparative genomic analysis and the mouse model revealed that Y18J is a nonpathogenic strain. The antibacterial secondary metabolites produced by Y18J are responsible for its antagonistic activity against the pathogenic ETEC strain. Our findings provide new insights into the molecular mechanism underlying gastrointestinal interactions between an indigenous bacterium and a pathogen from pigs. In the current environment of highly efficient animal production, our work promotes comparative genomics studies to clarify the genomic and biological diversity of E. coli strains.

## MATERIALS AND METHODS

### Bacterial screening and identification.

Feces samples were collected aseptically according to the instructions of consultants from experimental piglet farms (Changsha, China). Each stool sample was filtered, and the filtrate was diluted and spread on MacConkey agar plates supplemented with nystatin (10 μg/mL) and erythromycin (25 μg/mL) (71). Pink colonies were selected and further subjected to subculturing on Luria-Bertani (LB) agar to obtain pure bacterial cultures. DNA of the isolates was extracted, and 16S rRNA genes were amplified using the universal primers 27F (5′-AGAGTTTGATCMTGGCTCAG-3′) and 1492R (5′-GGTTACCTTGTTACGACTT-3′) (72) (Table S6). PCR was performed using the primeSTAR Max 2× premix kit (TaKaRa, no. R045Q) in a final volume of 50 μL. The program parameters were set as 98°C for 2 min, 35 cycles at 98°C for 10 s, annealing at 57°C for 10 s and 72°C for 30 s, and finally a hold at 16°C. The PCR amplified products were sequenced using Sanger sequencing (Sangon Biotech Ltd., China) and were analyzed against the nonredundant NCBI nucleotide collection database using BLASTN. The nearest match for each sequence was selected, and taxonomy was assigned at the species level.

### Genome sequencing, assembly, annotation, and comparison.

For Y18J or W25K, genomic DNA was obtained from 75 mL bacterial fermentation broth, using a commercial DNA extraction kit (catalog no. DP302; Tiangen, China) and a NEBNext Ultra DNA library preparation kit (New England Biolabs [NEB], USA; catalog no. E7645) for Illumina (NEB, USA) following the manufacturer’s recommendations. We utilized the hybrid assembly approach to sequence the genomes, using the short reads from Illumina HiSeq X Ten and long reads from PacBio Sequel. Raw reads were generated and processed using Trimmomatic (version 0.36) (73) to obtain clean reads. A SMRTbell template library was also constructed and sequenced on the PacBio Sequel platform. These nucleotide reads were subsequently used for *de novo* assembly using Falcon (74) with default settings, and the contigs were circularized using Circlator (75). To obtain a high-quality genome, the circularized and trimmed contigs were further polished with next-generation sequencing (NGS) short reads using Pilon (version 1.22) (76). The assembled genome was annotated using the NCBI Prokaryotic Genome Annotation Pipeline to find protein-coding genes and RNA genes.

Gene families present in both Y18J and ETEC W25K were defined with OrthoFinder (version 2.5.4) (77). Genome synteny of the two strains was detected with i-ADHoRe (version 3.0) (78), and the synteny plot was drawn with CIRCUS (Fig. 3A). The regions of homology between W25K and Y18J were defined as four types, according to the three divisions of average length of homologues regions, including 30 kb, 100 kb, and 500 kb, followed by annotation in the COG database (Fig. 3B).

### Comparative genomic analysis of E. coli strains.

Our team downloaded the sequence information of pathogens, including ETEC, APEC, UPEC, AIEC, NMEC, EAEC, StxEAEC, EHEC, and EPEC, and all probiotic E. coli strains from GenBank (Table S7). The Y18J genome had five copies of the 16S rRNA gene and only one copy of *gyrB*; thus, the phylogenetic trees were constructed by the neighbor-joining method with the *gyrB* genes using the Geneious version 11.1.4 software package. Putative virulence genes were identified using VFDB (31) with default parameters.

After functional annotations of predicted protein-coding sequences, proteins were classified according to the COG, GO, KEGG, and CAZymes databases. The distribution of genes in the different functional classifications was compared between Y18J and W25K strains. The presence of drug resistance genes was predicted using the CARD by discarding the loose hits (<95% identity) (26). The genomes of Y18J and W25K strains were analyzed for the presence of secondary-metabolite BGCs using antiSMASH (79).

### Bacterial growth curve.

E. coli strains were cultured in LB medium at 37°C overnight with shaking at 200 rpm. The overnight culture was incubated in 50 mL LB broth in baffled flasks at 37°C with shaking at 200 rpm. The optical density at 600 nm was measured every 30 min using a UV-1700 spectrophotometer. Each sample was analyzed in triplicate.

### Antimicrobial activity.

Antimicrobial activity assays were determined using the agar well diffusion assay (38). Briefly, E. coli strains were grown in LB broth at 37°C for 12 h. One microliter of W25K was used to flood an LB agar plate, which was then kept at room temperature for 40 min for drying. Wells were prepared in each agar plate by using a sterile iron pipette with a depth of 6 mm and a diameter of 5 mm. Precisely 30 μL of Y18J (wild type or mutant strain) culture was added to each well, and one remaining well was filled with 30 μL of LB broth as the negative control. The bacterial cultures were heat inactivated at 98°C for 10 min. The plates were then incubated under aerobic conditions at 37°C for 24 h, and the antibacterial effects were observed.

### Antibiotic susceptibility testing.

The susceptibility of E. coli to 26 antibiotics of seven different classes was also evaluated with disk diffusion tests (80). Bacterial fermentation liquid was inoculated into melted LB medium at a ratio of 10,000:1. After mixing and cooling for solidification, disks from an antibiotic kit (Hangzhou Microbial Reagent Co., Ltd.) (81) were added on the surface of the medium. Bacteria were aerobically incubated at 37°C for 12 h. The transparent inhibition zone diameters for each antibiotic were measured, and the results were compared with breakpoint values designated according to the product manual.

### Administration of E. coli strains by gavage in the mouse model.

The Animal Care and Use Committee of the Institute of Subtropical Agriculture, Chinese Academy of Sciences, reviewed and approved the experimental procedures. A total of 54 mice were randomly allocated to 3 groups with 3 replicate cages per treatment and 6 mice per cage, including a control group (phosphate-buffered saline, 2.0 mL each day for 5 days), a Y18J solution group (10^8^ CFU/mL, 2.0 mL each day for 5 days) and a W25K solution group (10^8^ CFU/mL, 2.0 mL each day for 5 days). The diarrhea rate was observed for 6 days after gavage and was calculated as total number of diarrheic mice per cage in 6 days/total number of mice per cage. SPSS software (version 19.0; IBM Corp., Chicago, IL, USA) was used to evaluate mouse diarrhea results with one-way analysis of variance and Duncan’s multiple-comparison test to determine the statistical significance of the differences among treatment groups.

### Recombineering for constructing Y18J mutants.

The recombinase expression plasmid pSC101-BAD-gbaA-hyg (82) (GeneBridges, Dresden, Germany) was electroporated into Y18J. The transformants were selected on LB plates supplemented with hygromycin (200 μg/mL). The Y18J electrocompetent cells were prepared according to our established protocol (83). Briefly, the DNA fragment with homology arms and the kanamycin resistance gene was amplified with the primers Colicin-km-loxM-3 and Colicin-km-loxM-5 from the plasmid pSC101-tetR-tetO-eGFP-km (84). From the overnight cultures of Y18J containing the recombinase expression plasmid pSC101-BAD-gbaA-hyg, 35 μL was inoculated into 1.4 mL LB medium with hygromycin (100 μg/mL). The cells were grown at 30°C at 900 rpm for 120 min in an Eppendorf ThermoMixer. After the induction of arabinose for the P_BAD_ promoter, the cells were incubated at 37°C for 45 min. Cells were then centrifuged for 30 s at 9,500 rpm and 4°C. The supernatant was discarded, and the cell pellets were resuspended in 1 mL ice-cold sterilized water and centrifuged. The ice-cold water washing was repeated once. Cells were resuspended in 30 μL ice-cold water, and 0.1 μg of DNA fragment was added. For recombineering, 400 ng PCR products with kanamycin resistance gene and homology arms were introduced into the electrocompetent Y18J cells using ice-cold cuvettes and an Eppendorf 2510 electroporator set at 1,250 V, 10 μF, and 600 Ω. After electroporation, cells were recovered in 1 mL fresh LB medium for 60 min. The recombinants were selected on the LB plates supplemented with kanamycin (30 μg/mL). The primers used for knockout of colicins are listed in Table S6.

### Recombineering for complementation of colicin B and colicin M.

The engineering of colicin B and colicin M complementation strains was based on the Y18J mutant. E. coli GB05-dir (GeneBridges, Dresden, Germany) competent cells were prepared, following a previously published method (82, 85). DNA fragments of colicin B and colicin M associated with their immunity genes were amplified by the primer pairs pGB-CliBI-hyg-3/pGB-CliBI-hyg-5 and pGB-CliMI-hyg-3/pGB-CliMI-hyg-5, respectively, from the Y18J genome. Then, the plasmid pGB-hyg-Ptet-cre (86) was digested with the restriction enzymes BamHI/HindIII, and the large fragment (the linear vector) was recovered by electrophoresis. When E. coli GB05-dir competent cells were ready, 2 ng of the linear vector and 2 ng of the DNA fragment of colicin B or colicin M were coelectroporated into competent cells with l-arabinose induction. The recombinants were cultured at 37°C and selected on the LB plates supplemented with ampicillin (100 μg/mL). The plasmids pGB-hyg-P_tet_-colicinBI and pGB-hyg-P_tet_-colicinMI were identified by enzyme digestion with NdeI and EcoRI. The correct clones were further verified by DNA sequencing. After sequencing verification, the complementation plasmids were electroporated into the Y18J mutant. The primers used for complementation of colicins are listed in Table S6.

### *In vitro* microbe-microbe interactions.

A GFP expression plasmid (pNCS-NeonGreen-amp) was first electroporated into strain W25K. Strains Y18J and W25K with the GFP expression plasmid were grown in monoculture for 12 to 16 h. Thirty microliters of each overnight monoculture was mixed and transferred into 1 mL fresh LB. One hundred microliters of coculture cell suspension was harvested at different coculture times, including the initial time, 6 h, and 12 h. The bacterial cells were diluted 10-fold in 5 orders of magnitude and then were plated on LB plates. Colony numbers with and without fluorescence were counted after incubation 24 h at 37°C. Data were statistically analyzed by using Student's *t* test with SPSS software. Each sample was analyzed in triplicate.

### Abundances of Y18J and W25K in metagenome.

Pig gut metagenome data were downloaded from China National GenBank DataBase with accession code CNP0000824. After comparison of the genomes of W25K and Y18J (Fig. 3A), it was found that the genes W_1 and W_2 are unique to W25K and Y_1 and Y_2 are unique to Y18J. These four genes in gut metagenomes of boars (*n* = 8), Duroc pigs (*n* = 7), and Laiwu pigs (*n* = 6) were analyzed by trough genome-wide alignment. Salmon (version 0.9.1) was used to quantify the abundance of each nonredundant metagenome-assembled genome with the four selected genes in each sample. The abundances were normalized to TPM (87). TPM of all samples were nonlinearly normalized by the formula *x* = log_2_(TPM + 0.5)/log_2_(max TPM + 0.5). About 6 to 8 samples were selected for each pig breed, including all 6 samples from Laiwu pigs, 8 of 14 samples from boars, and 7 of 10 samples from Duroc pigs, to undergo the following steps. In order to make the numbers of samples for the three pigs almost identical, we screened the samples of Duroc pigs and boars to make their numbers almost identical to those from Laiwu pigs. In the first screening step, the five samples with positive expression of the four genes were selected for Duroc pigs and boars. In the second screening step, by Spearman’s nonparametric correlation analysis and clustering visualization, we added Boar_s18, Boar_s19, Boar_s35, Duroc_Reads.High3, and Duroc_Reads.Low2 to the results from the previous step for Duroc pigs and boars, as they have high correlation coefficients with the results of the first-screening samples. Red columns in the heat map of Spearman’s coefficient represent high similarity. A violin plot was drawn with Prism 8. A difference test between Y18J (Y_1 and Y_2) and W25K (W_1 and W_2) was verified by Mann-Whitney U test (R version 3.5).

### Data availability.

The complete genome of E. coli Y18J has been deposited in GenBank with accession numbers CP076293, CP076294, CP076295, and CP076296 for the chromosome, plasmid 1, plasmid 2, and plasmid 3, respectively. The complete genome of enterotoxigenic E. coli W25K has also been deposited in GenBank with accession numbers CP091038.1, CP091042.1, CP091041.1, CP091040.1, and CP091039.1 for the chromosome, plasmid 1, plasmid 2, plasmid 3, and plasmid 4, respectively.
