# Supplementary material for: Colicins of Escherichia coli Lead to Resistance against the Diarrhea-Causing Pathogen Enterotoxigenic E. coli in Pigs
Source: Microbiol Spectr. 2022 Oct 3;10(5):e01396-22. doi: 10.1128/spectrum.01396-22 (PMC9603048; doi:10.1128/spectrum.01396-22)
Supplement: Supplemental file 8 — Fig. S1 to S10 and Tables S1 to S7. Download spectrum.01396-22-s0008.pdf, PDF file, 2.9 MB [file spectrum.01396-22-s0008.pdf]

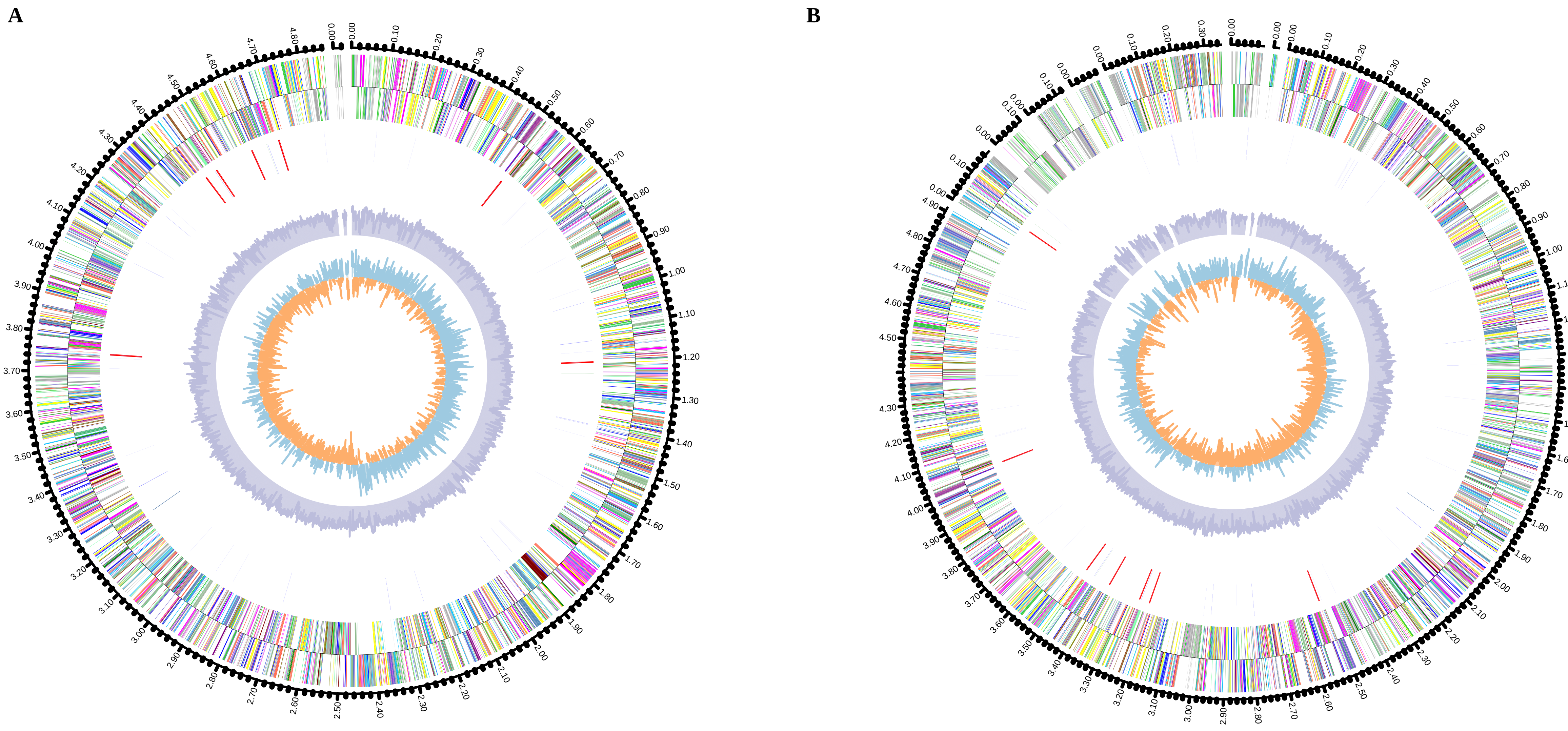

Figure S1. Circular maps of the Y18J (A) and W25K (B). Gene map from outside to inside: circle 1, the size of chromosome; circle 2 and circle 3, the predicted protein-coding genes on the + and − strands, different colors represent different COG functional classifications; circle 4, rRNA and tRNA; circle 5, G + C content; the innermost circle, G + C skew.

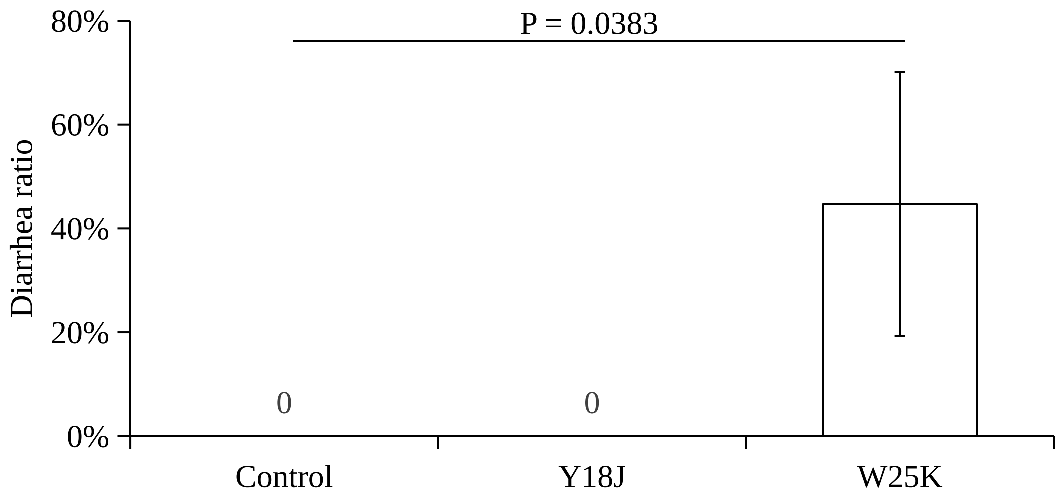

Figure S2. Mice diarrhea rate after gavage of Y18J and W25K strains. For control phosphate-buffered saline was used. Data are means of 3 replicate cages with 6 mice per treatment.

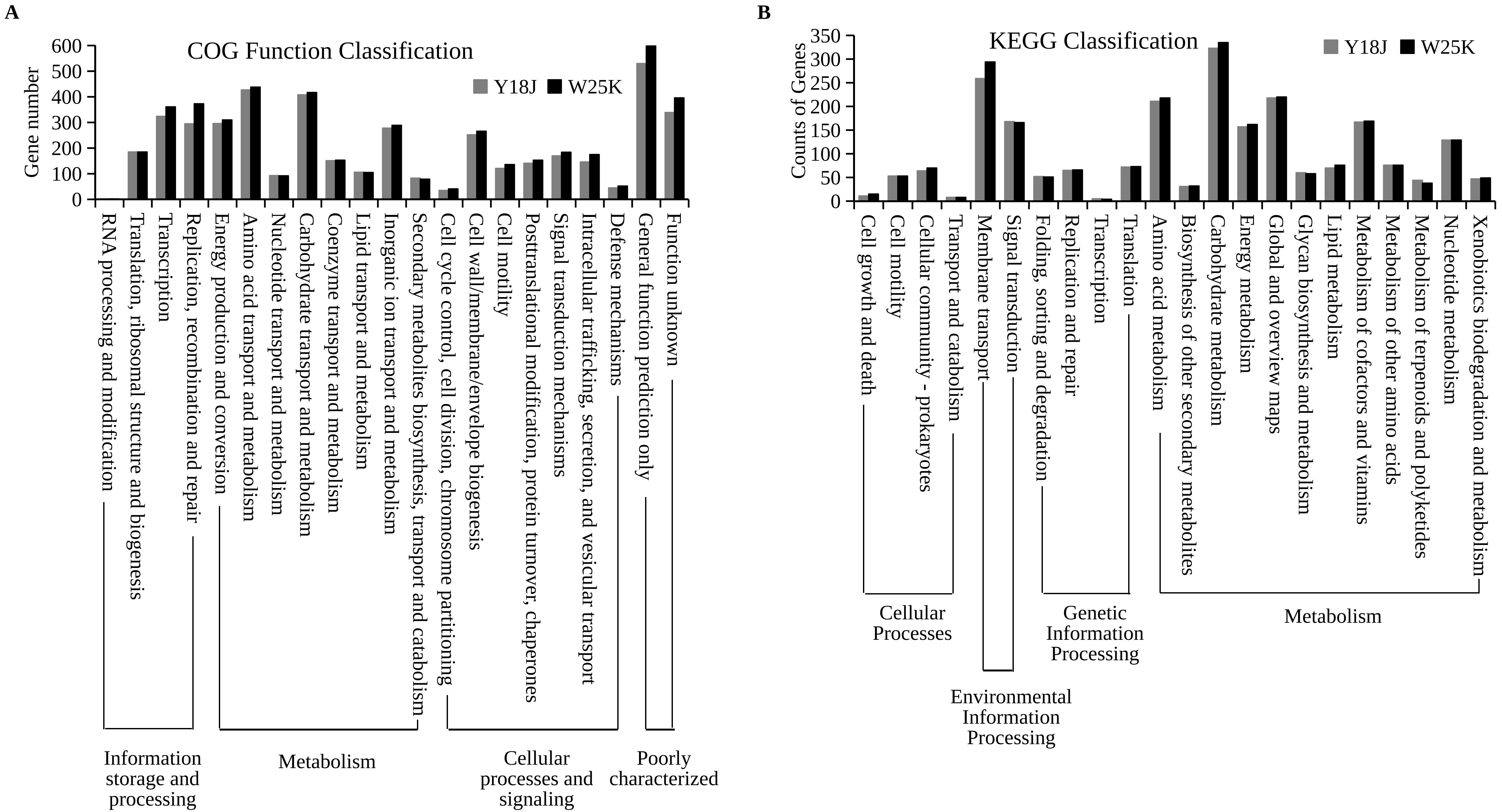

Figure S3. Comparative functional annotation and analysis of the Y18J and W25K genome. (A) COG function annotation. The COG function is further divided into 4 parts: information storage and processing, metabolism, cellular processes and signaling, and poorly characterized. (B) Gene number of KEGG pathways in Y18J and W25K. The KEGG classification is further divided into four categories: cellular processes, environmental information processing, genetic information processing, and metabolism.

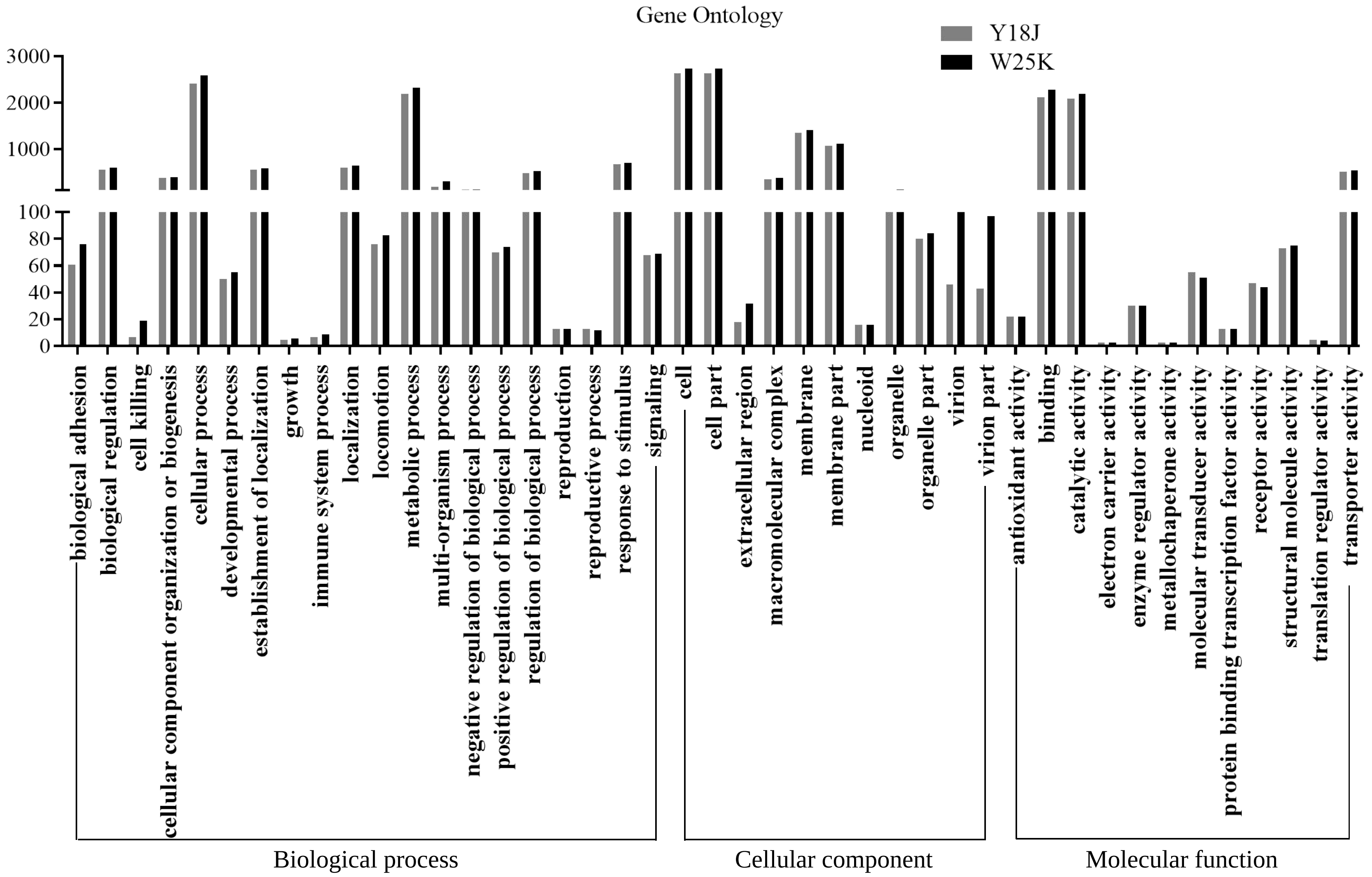

Figure S4. The gene number of GO-terms in Y18J and W25K.

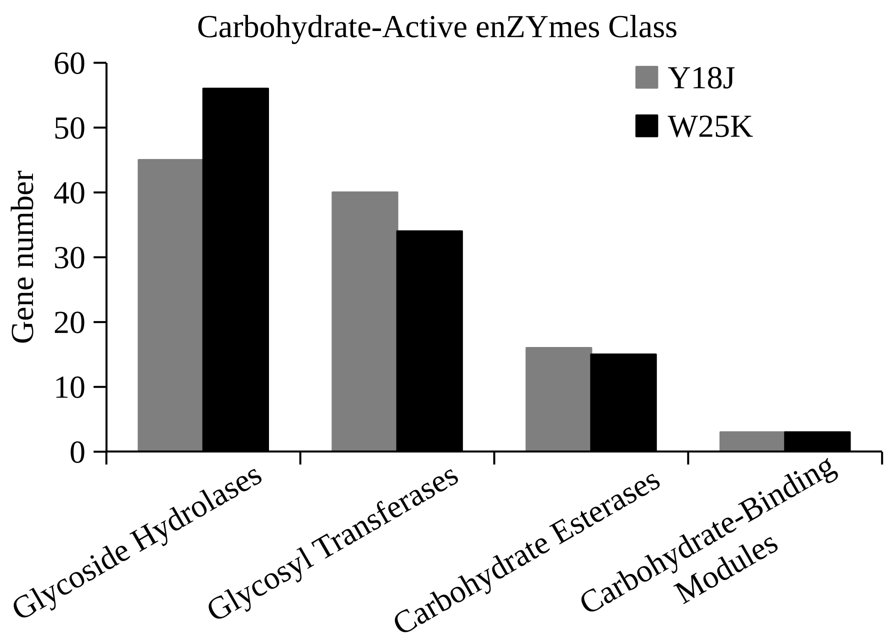

Figure S5. Gene numbers of CAZymes in Y18J and W25K.

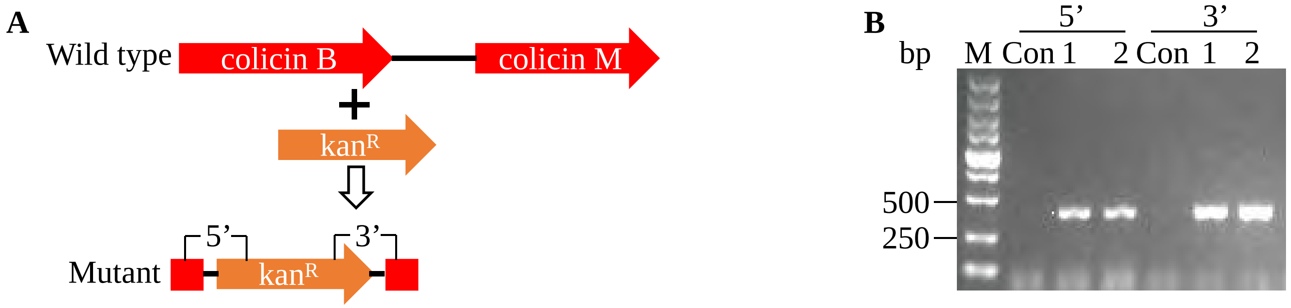

Figure S6. Diagram showing the construction and verification of BGC1 inactivation in Y18J. (A) Diagram showing the construction of BGC1 inactivation (Mutant) in Y18J using Red/ET recombineering. (B) PCR verification of the inactivation of BGC1. Control (Con) is Y18J wild type strain, lane 1 and 2 are correct clones after recombineering.

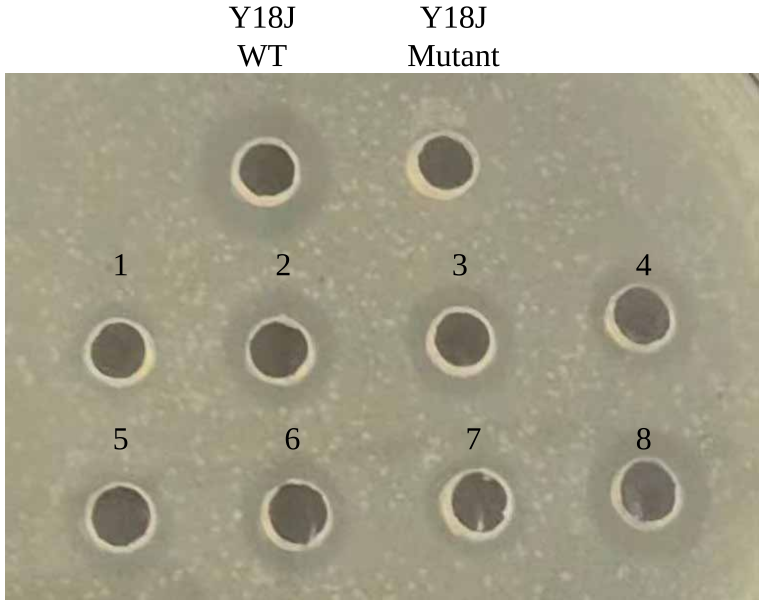

Figure S7. Inhibitory effects of colicin B-complementation (clones 1-4) and colicin M-complementation (clones 5-8) in Y18J mutant strain against the pathogen W25K.

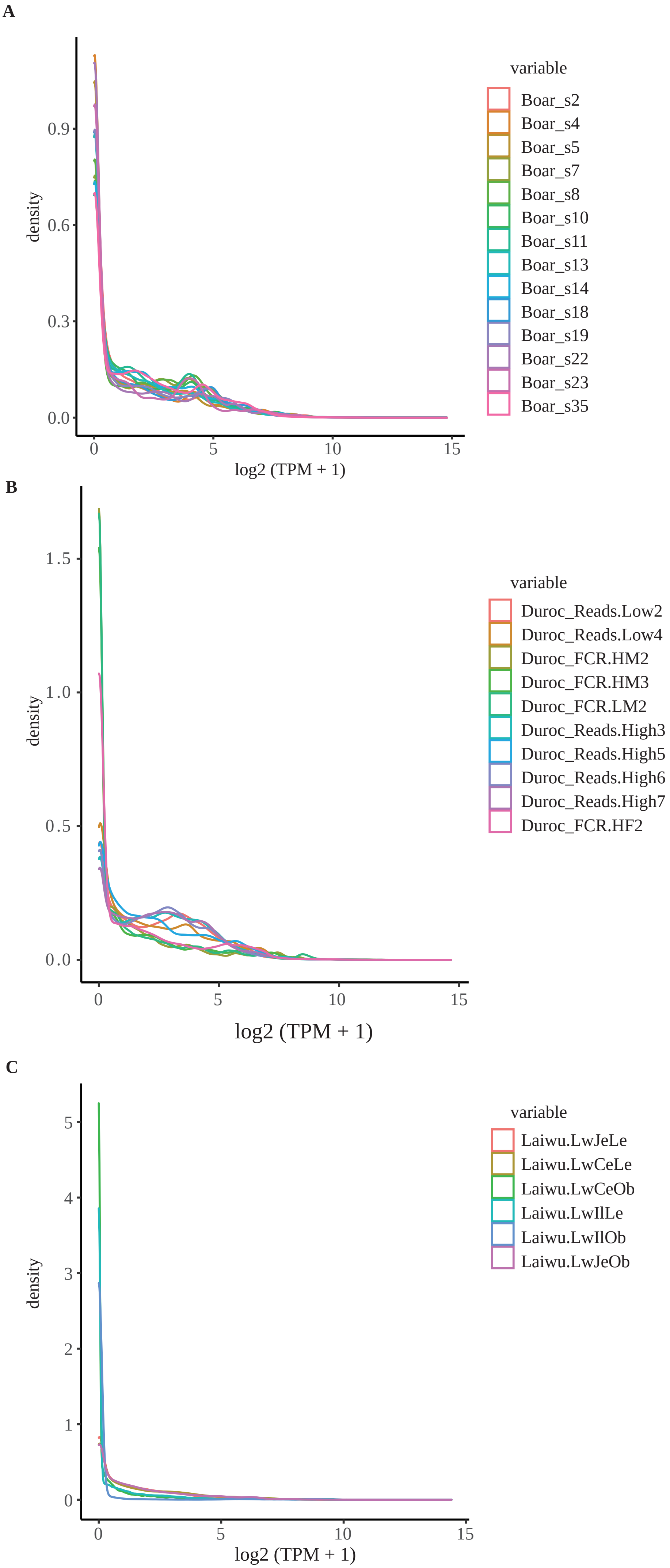

Figure S8. TPM distributions of the three pigs. All samples are fitted normal distribution after log transformation in Boars (A), Duroc pigs (B) and Laiwu pigs(C) by using shapiro.test in R.

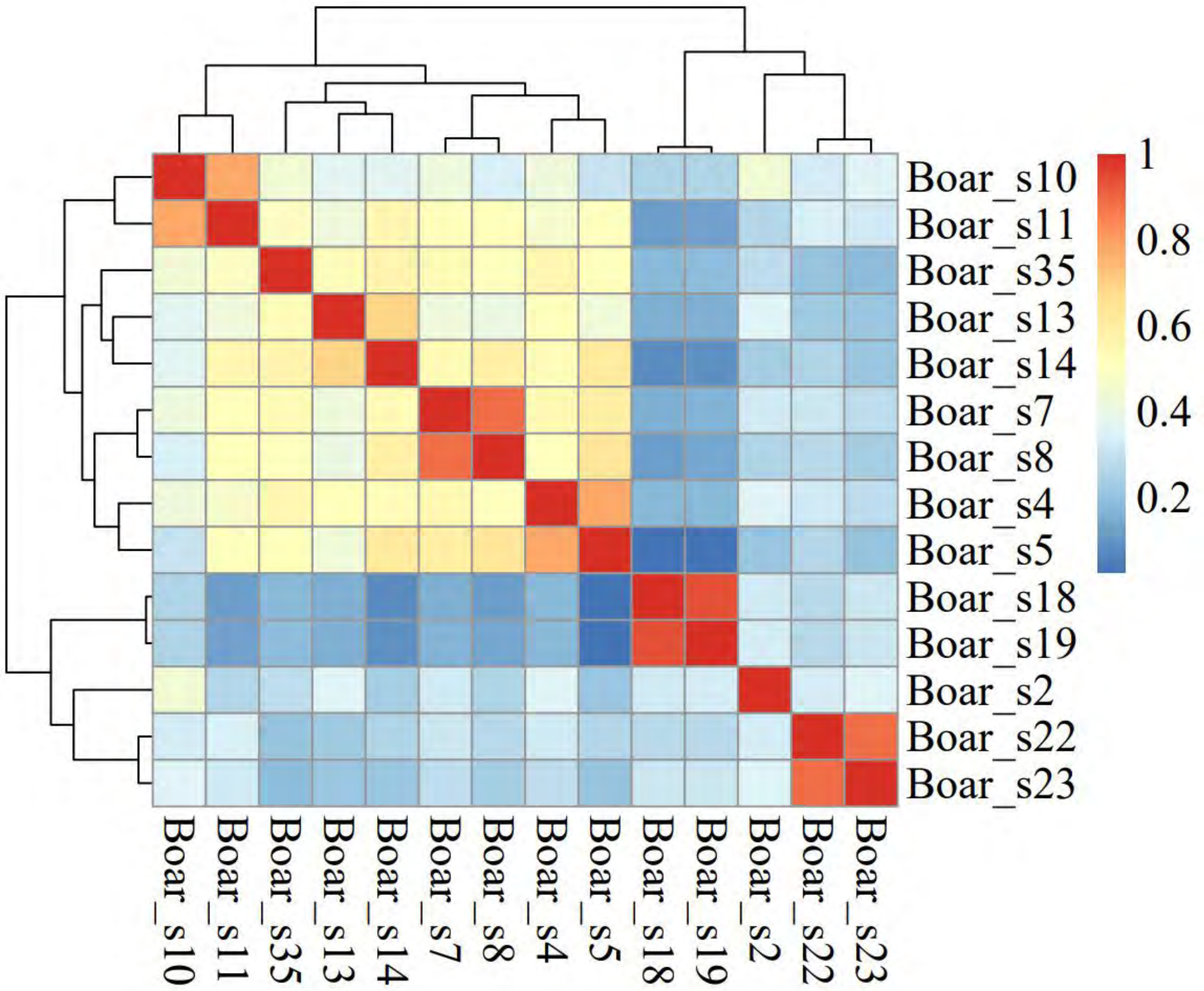

Boar_s13

Boar_s14

Boar_s18

Boar_s19

Boar_s2

Boar_s22

Boar_s23

Boar_s35

Figure S9. Samples selection for Boars. Samples with bold name were selected at first because they have positive TPM value for any of the four genes (W_1, W_2, Y_1, Y_2). Based on the correlation analysis among these samples (Spearman's non-parametric correlation), the other 3 samples were also selected to guarantee that the total number was around 7, which was the sample number of Laiwu pigs.

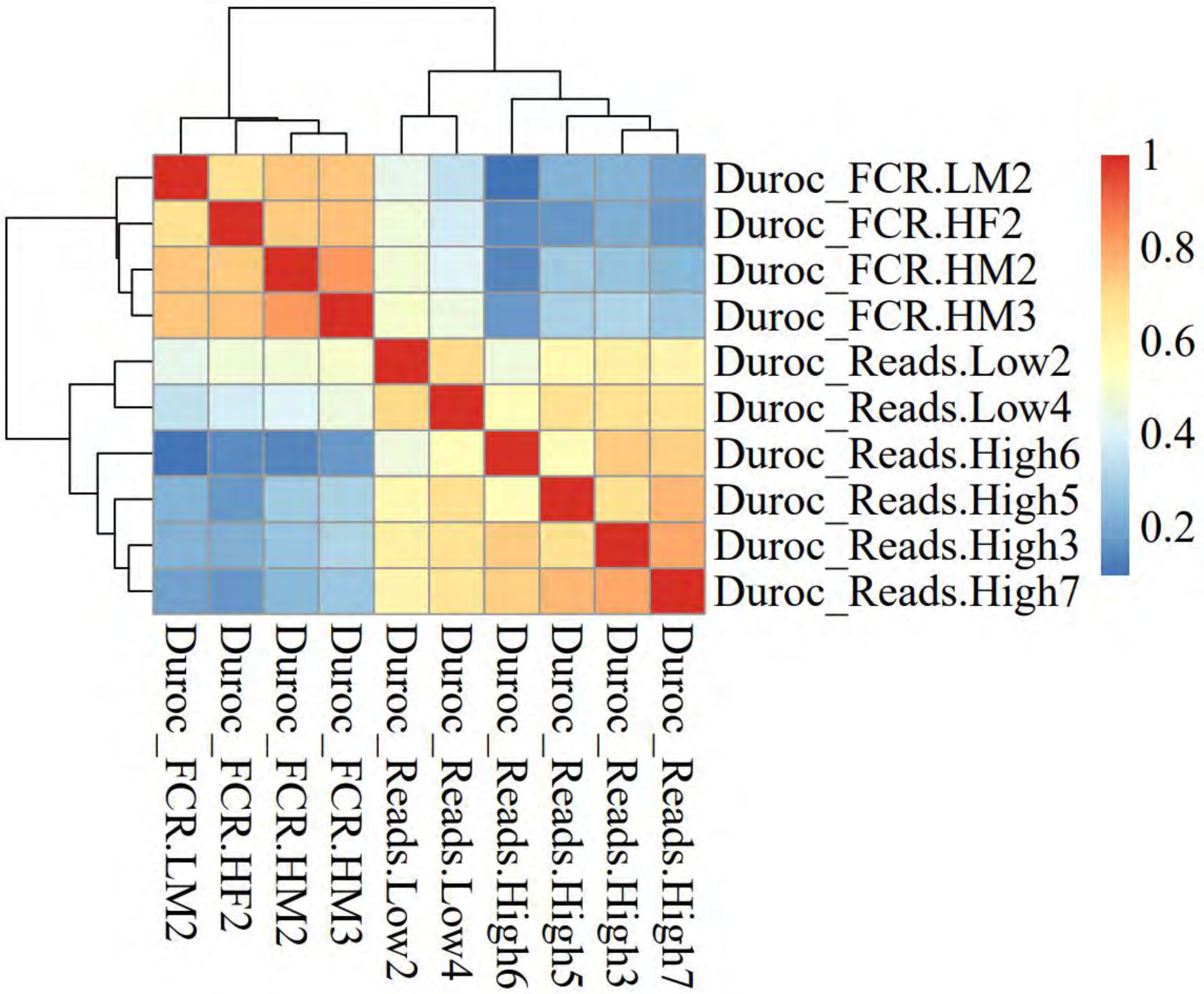

Duroc_FCR.HM2

Duroc_FCR.HM3

Duroc_Reads.High3

Duroc_Reads.High5

Duroc_Reads.High7

Duroc_Reads.Low2

Duroc_Reads.Low4

Figure S10. Samples selection for Duroc pigs. Samples with bold name were selected at first as they have positive TPM value for any of the four genes (W_1, W_2, Y_1, Y_2). Based on the correlation analysis among these samples (Spearman's non-parametric correlation), the other 2 samples were also selected to guarantee that the total number was around the Laiwu sample number 7.

TABLE S1. The sensitivity of Y18J against 26 antibiotics, and drug-resistance genes present in its genome

Types	Drug	μg/pill	The zone of inhibition[a]	Antibiotic resistance gene[b]
Penicillins	**Penicillin**	100	0 (R)	gene3985: *CMY-47*
	**Oxacillin**	1	0 (R)	gene3985: *CMY-47*
	**Carboxycillin**	100	0 (R)	gene3985: *CMY-47*
	**Ampicillin**	10	0 (R)	gene3985: *CMY-47*
Cephalosporins	Cephalexin	30	2.3 (S)	
	Cefazolin	30	2.8 (S)	
	Cefradine	30	2.0 (I)	
	Ceftazidime	30	3.1 (S)	
	Cefoperazone	75	2.8 (S)	
Aminoglycosides	Amikacin	30	1.7 (I)	
	Kanamycin	30	2.2 (S)	
	**Neomycin**	30	1.3 (R)	
	Gentamycin	15	2.8 (S)	
	**Spectinomycin**	80	0 (R)	
	Hygromycin	200	2.9 (S)	
Tetracyclines	**Tetracycline**	30	0 (R)	
	**Doxycycline**	30	1.1 (R)	
Macrolides	**Erythromycin**	15	0 (R)	gene1919: *msrB*
	**Midecamycin**	30	0 (R)	
Glycopeptides	**Vancomycin**	3	0 (R)	
Quinolones	**Norfloxacin**	10	0 (R)	gene1712: *mdtK*
	**Ofloxacin**	5	0 (R)	
	**Ciprofloxacin**	5	0 (R)	
	Furazolidone	300	2.1 (I)	
	**Chloramphenicol**	30	0 (R)	gene2926: *mdfA*
	**Clindamycin**	2	0 (R)	

[a]The zone of inhibition (diameter in cm) for each antibiotic was measured and expressed as susceptible, S (≥2.2 cm); intermediate, I (1.4–2. 1 cm), and resistance, R (<=1.3 cm). Resistant drugs were displayed in bold font.

[b]Antibiotic resistance genes were identified using the CARD database.

TABLE S2. The sensitivity of W25K against 26 antibiotics, and drug-resistance genes present in its genome

Types	Drug	μg/pill	The zone of inhibition[a]	Antibiotic resistance gene[b]
Penicillins	**Penicillin**	100	0 (R)	gene2957: *CMY-47*
	**Oxacillin**	1	0 (R)	gene2957: *CMY-47*
	Carboxycillin	100	2.2 (S)	
	Ampicillin	10	1.4 (I)	
Cephalosporins	Cephalexin	30	2.2 (S)	
	Cefazolin	30	1.6 (I)	
	Cefradine	30	1.4 (I)	
	Ceftazidime	30	1.4 (I)	
	Cefoperazone	75	2.4 (S)	
Aminoglycosides	**Amikacin**	30	0.8 (R)	gene3463: *aadA*; gene5438: *acrD*
	**Kanamycin**	30	0.6 (R)	gene3463: *aadA*; gene5438: *acrD*
	Neomycin	30	2.4 (S)	
	**Gentamycin**	15	0.6 (R)	gene3463: *aadA*; gene5438: *acrD*
	**Spectinomycin**	80	0 (R)	gene3463: *aadA*; gene5438: *acrD*
	**Hygromycin**	200	0 (R)	gene3463: *aadA*; gene5438: *acrD*
Tetracyclines	**Tetracycline**	30	0 (R)	gene5253: *tetA*; gene5255: *tetD*
	**Doxycycline**	30	0 (R)	gene5253: *tetA*; gene5255: *tetD*
Macrolides	**Erythromycin**	15	0 (R)	gene1163: *MsrB*; gene1319: *emrE*
	**Midecamycin**	30	0 (R)	
Glycopeptides	**Vancomycin**	3	0.4 (R)	
Quinolones	Norfloxacin	10	1.8 (I)	
	**Ofloxacin**	5	0 (R)	
	Ciprofloxacin	5	2.0 (I)	
	**Furazolidone**	300	0 (R)	
	Chloramphenicol	30	1.8 (I)	
	**Clindamycin**	2	0 (R)	

[a]The zone of inhibition (diameter in cm) for each antibiotic was measured and expressed as susceptible, S (≥2.2 cm); intermediate, I (1.4–2. 1 cm), and resistance, R (<=1.3 cm). Resistant drugs were displayed in bold font.

[b]Antibiotic resistance genes were identified using the CARD database.

TABLE S3. Chromosome and plasmids feature for Y18J and W25K

Sample	Genome_size (bp)	Gene_ num	Total_len (bp)	Average_len (bp)	GC% (gene region)	ncRNA genes	Gene_len/ Genome(%)	Accession number
Y18J								
Chromosome (y1)	4883445	4557	4285032	940.32	51.95	118	87.75	CP076293
Plasmid 1 (y2)	47380	51	41691	817.47	52.25	0	87.99	CP076294
Plasmid 2 (y3)	30661	38	22188	583.89	51.69	0	72.37	CP076295
Plasmid 3 (y4)	20482	27	13143	486.78	49.99	0	64.17	CP076296
W25K								
Assemble contigs (w1-4)	5457385	5249	4773294	909.37	51.76	123	87.46	JANPYO000000000
Plasmid 1 (w5)	126198	146	106557	729.84	50.88	0	84.44	CP091042.1
Plasmid 2 (w6)	100605	122	80085	656.43	52.25	0	79.6	CP091041.1
Plasmid 3 (w7)	97720	110	87486	795.33	48.03	3	89.53	CP091040.1
Plasmid 4 (w8)	80970	101	65331	646.84	50.37	0	80.69	CP091039.1

TABLE S4. The drug-resistance genes present in Y18J strain

Query_seq	Resistance Gene	Description
gene0055	*vgaC*	VgaC is an efflux protein expressed in *Staphylococci* that confers resistance to streptogramin A antibiotics and related compounds. It is associated with plasmid DNA.
gene0237	*gadX*	GadX is an AraC-family regulator that promotes mdtEF expression to confer multidrug resistance.
gene0238	*gadW*	GadW is an AraC-family regulator that promotes mdtEF expression to confer multidrug resistance. GadW inhibits GadX-dependent activation. GadW clearly represses gadX and, in situations where GadX is missing, activates gadA and gadBC.
gene0242	*mdtF*	MdtF is the multidrug inner membrane transporter for the MdtEF-TolC efflux complex.
gene0243	*mdtE*	MdtE is the membrane fusion protein of the MdtEF multidrug efflux complex. It shares 70% sequence similarity with AcrA.
gene0425	*CRP*	CRP is a global regulator that represses MdtEF multidrug efflux pump expression.
gene0509	*acrF*	AcrF is a inner membrane transporter, similar to AcrB.
gene0510	*acrE*	AcrE is a membrane fusion protein, similar to AcrA.
gene0511	*acrS*	AcrS is a repressor of the AcrAB efflux complex and is associated with the expression of AcrEF. AcrS is believed to regulate a switch between AcrAB and AcrEF efflux.
gene0701	*patA*	PatA is an ABC transporter of *Streptococcus pneumoniae* that interacts with PatB to confer fluoroquinolone resistance.
gene0716	*bacA*	BacA is a gene that recycles undecaprenyl pyrophosphate during cell wall biosynthesis which confers resistance to bacitracin.
gene0736	*tolC*	TolC is a protein subunit of many multidrug efflux complexes in Gram negative bacteria. It is an outer membrane efflux protein and is constitutively open. Regulation of efflux activity is often at its periplasmic entrance by other components of the efflux complex.
gene0937	*dfrE*	DfrE is a chromosome-encoded dihydrofolate reductase found in *Enterococcus faecalis*.
gene1075	*emrB*	EmrB is a translocase in the emrB -TolC efflux protein in *E. coli*. It recognizes substrates including carbonyl cyanide m-chlorophenylhydrazone (CCCP), nalidixic acid, and thioloactomycin.
gene1076	*emrA*	EmrA is a membrane fusion protein, providing an efflux pathway with EmrB and TolC between the inner and outer membranes of *E. coli*, a Gram-negative bacterium.
gene1077	*emrR*	EmrR is a negative regulator for the EmrAB-TolC multidrug efflux pump in *E. coli*. Mutations lead to EmrAB-TolC overexpression.
gene1259	*acrD*	AcrD is an aminoglycoside efflux pump expressed in *E. coli*. Its expression can be induced by indole, and is regulated by baeRS and

		cpxAR.
gene1343	*evgS*	EvgS is a sensor protein that phosphorylates the regulatory protein EvgA. evgS corresponds to 1 locus in *Pseudomonas aeruginosa* PAO1 and 1 locus in *Pseudomonas aeruginosa* LESB58.
gene1344	*evgA*	EvgA, when phosphorylated, is a positive regulator for efflux protein complexes emrKY and mdtEF. While usually phosphorylated in a EvgS dependent manner, it can be phosphorylated in the absence of EvgS when overexpressed.
gene1345	*emrK*	EmrK is a membrane fusion protein that is a homolog of EmrA. Together with the inner membrane transporter EmrY and the outer membrane channel TolC, it mediates multidrug efflux.
gene1346	*emrY*	EmrY is a multidrug transport that moves substrates across the inner membrane of the Gram-negative *E. coli*. It is a homolog of emrB.
gene1445	*arnA*	ArnA modifies lipid A with 4-amino-4-deoxy-L-arabinose (Ara4N) which allows gram-negative bacteria to resist the antimicrobial activity of cationic antimicrobial peptides and antibiotics such as polymyxin. arnA is found in *E. coli* and *P. aeruginosa*.
gene1446	*pmrF*	PmrF is required for the synthesis and transfer of 4-amino-4-deoxy-L-arabinose (Ara4N) to Lipid A, which allows gram-negative bacteria to resist the antimicrobial activity of cationic antimicrobial peptides and antibiotics such as polymyxin. pmrF corresponds to 1 locus in *Pseudomonas aeruginosa* PAO1 and 1 locus in *Pseudomonas aeruginosa* LESB58.
gene1491	*yojI*	YojI mediates resistance to the peptide antibiotic microcin J25 when it is expressed from a multicopy vector. YojI is capable of pumping out microcin molecules. The outer membrane protein TolC in addition to YojI is required for export of microcin J25 out of the cell. Microcin J25 is thus the first known substrate for YojI.
gene1615	*baeR*	BaeR is a response regulator that promotes the expression of MdtABC and AcrD efflux complexes.
gene1616	*baeS*	BaeS is a sensor kinase in the BaeSR regulatory system. While it phosphorylates BaeR to increase its activity, BaeS is not necessary for overexpressed BaeR to confer resistance.
gene1618	*mdtC*	MdtC is a transporter that forms a heteromultimer complex with MdtB to form a multidrug transporter. MdtBC is part of the MdtABC-TolC efflux complex. In the absence of MdtB, MdtC can form a homomultimer complex that results in a functioning efflux complex with a narrower drug specificity. mdtC corresponds to 3 loci in *Pseudomonas aeruginosa* PAO1 (gene name: muxC/muxB) and 3 loci in *Pseudomonas aeruginosa* LESB58.
gene1619	*mdtB*	MdtB is a transporter that forms a heteromultimer complex with MdtC to form a multidrug transporter. MdtBC is part of the MdtABC-TolC efflux complex.
gene1620	*mdtA*	MdtA is the membrane fusion protein of the multidrug efflux complex mdtABC.
gene1666	*pmrE*	PmrE is required for the synthesis and transfer of 4-amino-4-deoxy-L-arabinose (Ara4N) to Lipid A, which allows gram-negative bacteria to resist the antimicrobial activity of cationic antimicrobial peptides and antibiotics such as polymyxin.

gene1712	*mdtK*	A multidrug and toxic compound extrusions (MATE) transporter conferring resistance to norfloxacin, doxorubicin and acriflavine.
gene1785	*sdiA*	SdiA is a cell division regulator that is also a positive regulator of AcrAB only when it's expressed from a plasmid. When the sdiA gene is on the chromosome, it has no effect on expression of acrAB.
gene1919	*msrB*	MsrB is an ABC-efflux pump expressed to *Staphylococcus* species that confers resistance to erythromycin and streptogramin B antibiotics. It is associated with plasmid DNA.
gene2245	*marA*	In the presence of antibiotic stress, *E. coli* overexpresses the global activator protein MarA, which besides inducing MDR efflux pump AcrAB, also down- regulates synthesis of the porin OmpF.
gene2511	*H-NS*	H-NS is a histone-like protein involved in global gene regulation in Gram-negative bacteria. It is a repressor of the membrane fusion protein genes acrE, mdtE, and emrK as well as nearby genes of many RND-type multidrug exporters.
gene2614	*Mfd*	Mfd dissociates transcription elongation complexes blocked at nonpairing lesions and mediates recruitment of DNA repair proteins. The Mfd (mutation frequency decline) protein, also known as transcription-repair coupling factor, is responsible for ATP-dependent removal of stalled RNA polymerase from DNA lesions by inducing forward movement of the RNA polymerase and subsequent recruitment of nucleotide excision repair machinery to the sites of the lesions.
gene2662	*mdtH*	Multidrug resistance protein MdtH.
gene2674	*mdtG*	The mdtG protein, also named YceE, appears to be a member of the major facilitator superfamily of transporters, and it has been reported, when overexpressed, to increase fosfomycin and deoxycholate resistances. mdtG is a member of the marA-soxS-rob regulon.
gene2807	*msbA*	MsbA is a multidrug resistance transporter homolog from *E. coli* and belongs to a superfamily of transporters that contain an adenosine triphosphate (ATP) binding cassette (ABC) which is also called a nucleotide-binding domain (NBD). MsbA is a member of the MDR-ABC transporter group by sequence homology. MsbA transports lipid A, a major component of the bacterial outer cell membrane, and is the only bacterial ABC transporter that is essential for cell viability.
gene2926	*mdfA*	Multidrug efflux pump in *E. coli*. This multidrug efflux system was originally identified as the Cmr/CmlA chloramphenicol exporter.
gene3067	*kdpE*	KdpE is a transcriptional activator that is part of the two-component system KdpD/KdpE that is studied for its regulatory role in potassium transport and has been identified as an adaptive regulator involved in the virulence and intracellular survival of pathogenic bacteria. kdpE regulates a range of virulence loci through direct promoter binding.
gene3249	*rosA*	RosA is part of an efflux pump/potassium antiporter system (RosAB) in Yersinia that confers resistance to cationic antimicrobial peptides such as polymyxin B.

gene3250	*rosB*	RosB is part of an efflux pump/potassium antiporter system (RosAB) in Yersinia that confers resistance to cationic antimicrobial peptides such as polymyxin B.
gene3266	*acrB*	Protein subunit of AcrA-AcrB-TolC multidrug efflux complex. AcrB functions as a herterotrimer which forms the inner membrane component and is primarily responsible for substrate recognition and energy transduction by acting as a drug/proton antiporter.
gene3469	*tet34*	Tet34 causes the activation of Mg2+-dependent purine nucleotide synthesis, which protects the protein synthesis pathway. It is found in Gram-negative *Vibrio*.
gene3814	*mdtM*	Multidrug resistance protein MdtM.
gene3985	*CMY-47*	CMY-47 is a beta-lactamase. From the Lahey list of CMY beta-lactamases.
gene4025	*pmrC*	PmrC mediates the modification of Lipid A by the addition of 4-amino-4-deoxy-L-arabinose (L-Ara4N) and phosphoethanolamine, resulting in a less negative cell membrane and decreased binding of polymyxin B.
gene4058	*mdtN*	Multidrug resistance efflux pump. Could be involved in resistance to puromycin, acriflavine and tetraphenylarsonium chloride.
gene4059	*mdtO*	Multidrug resistance efflux pump. Could be involved in resistance to puromycin, acriflavine and tetraphenylarsonium chloride.
gene4060	*mdtP*	Multidrug resistance efflux pump. Could be involved in resistance to puromycin, acriflavine and tetraphenylarsonium chloride.
gene4223	*cpxA*	CpxA is a membrane-localized sensor kinase that is activated by envelope stress. It starts a kinase cascade that activates CpxR, which promotes efflux complex expression.
gene4412	*mdtL*	Multidrug resistance protein MdtL.
gene4448	*emrD*	EmrD is a multidrug transporter from the Major Facilitator Superfamily (MFS) primarily found in *Escherichia coli*. EmrD couples efflux of amphipathic compounds with proton import across the plasma membrane.

TABLE S5. BGCs predicted in Y18J and W25K by antiSMASH 5.0.

BGC	Type	From	To	Most similar known cluster	Similarity
Y18J					
Cluster 1	RiPP-like	55,071	66,606	microcin L	18%
Cluster 2	NRPS, T1PKS	1,848,976	1,907,159	yersiniabactin	35%
Cluster 3	Thiopeptide	2,998,183	3,024,475	O-antigen	14%
Cluster 4	NRPS	3,354,925	3,408,448	turnerbactin	30%
W25K					
Cluster 1	Thiopeptide	1,674,395	1,700,687	colicin V	1%
Cluster 2	NRPS	2,080,450	2,124,331	turnerbactin	30%

TABLE S6. Primers used in this study

Primer	Primer sequence (5'-3')	Application
27F	AGAGTTTGATCMTGGCTCAG	Bacterial identification
1492R	GGTTACCTTGTTACG ACTT	
Colicin-km-loxM-3	ATGTACAGTATTTAATTTTAATTGATTGTTTTTAAAGTCAAAGAGGTTTTCAACTTAAATGT GAAAGTGGGTC	Knocking out the colicin gene cluster
Colicin-km-loxM-5	TGTCATTAAATATTTTAAAGGGAAGCAGTAACACTGCCTTCCTTTTAATTAGCTTTAATGC GGTAGTTTATC	
pGB-CliBI-hyg-3	GCACAATGTGCGCCATTTTTCACTTCACAGGTCAAGCTTCTTATATTATCGCTTACCAC	Colicin B complementation
pGB-CliBI-hyg-5	AATGAATAGTTCGACAAAAATCTAGCAGGAGGAATTCATATGAGTGATAATGAAGGTAG	
pGB-CliMI-hyg-3	GCACAATGTGCGCCATTTTTCACTTCACAGGTCAAGCTTCTGCAATATGTGATCTCTTG	Colicin M complementation
pGB-CliMI-hyg-5	ATAGTTCGACAAAAATCTAGCAGGAGGAATTCATATGGAAACCTTAACGGTTCATG	

TABLE S7. Strains and plasmids in this work

E. coli strain	Type	Host/Source	Accession	Description
Y18J	-----	Pig	CP076293, CP076294, CP076295, CP076296	-----
W25K	ETEC	Pig	CP091038.1, CP091039.1, CP091040.1, CP091041.1, CP091042.1	-----
Nissle 1917	Probiotic	Human	NZ_CP007799.1, NZ_CP023342.1, NZ_MW240712.1	Phylogeny and virulence factors analysis
ABU 83972	Probiotic	Human	NC_017631.1, NC_017629.1	Phylogeny and virulence factors analysis
APEC O1	APEC	Turkey	NC_008563.1, NC_009837.1, NC_009838.1	Phylogeny and virulence factors analysis
UPEC 536	UPEC	Human	NC_008253.1	Phylogeny and virulence factors analysis
AIEC LF82	AIEC	---	NC_011993.1, NC_011917.1	Phylogeny and virulence factors analysis
NMEC O7:K1	NMEC	Human	NC_017646.1, NC_017647.1, NC_017648.1, NC_017649.1	Phylogeny and virulence factors analysis
EAEC 55989	EAEC	Human	NZ_CP028304.1, NC_011752.1	Phylogeny and virulence factors analysis
StxEAEC O104:H4	StxEAEC	Human	CP003301.1, NC_018663.1, NC_018662.1	Phylogeny and virulence factors analysis
ETEC UMNK88	ETEC	Pig	NC_017641.1, NC_017643.1, NC_017645.1	Phylogeny and virulence factors analysis
EHEC O157:H7	EHEC	---	NC_002695.2, AB011549.2, AB011548.2	Phylogeny and virulence factors analysis
EPEC O55:H7	EPEC	Human	NC_013941.1, CP001847.1,	Phylogeny and virulence factors analysis
G4/9	Probiotic	Human	JPKJ00000000	Phylogeny and virulence factors analysis
G5	Probiotic	Human	JPKK01000001.1, KM107843, KM107844,	Phylogeny and virulence factors analysis

			KM107845, KM107847	
G3/10	Probiotic	Human	JPKI00000000, JN887338, KM107838, KM107839, KM107840, KM107841, KM107842	Phylogeny and virulence factors analysis
Y18J mutant	---	---	---	The colicin B and M were knocked out
Plasmids				
pGB-hyg-Ptet-colicinBI	---	---	---	The colicin B and its immunity protein are under the tetracyclines inducible promoter
pGB-hyg-Ptet-colicinMI	---	---	---	The colicin M and its immunity protein are under the tetracyclines inducible promoter
pNCS-NeonGreen-amp	---	---	---	*ampR*, Green fluorescence
pSC101-BAD-gbaA-hyg	---	---	---	*hygR*, *redγβα* under BAD promoter
pGB-hyg-Ptet-cre	---	---	---	*hygR* under tetracycline-inducible promoter
